# Structural basis for potent antibody neutralization of SARS-CoV-2 variants including B.1.1.529

**DOI:** 10.1126/science.abn8897

**Published:** 2022-03-24

**Authors:** Tongqing Zhou, Lingshu Wang, John Misasi, Amarendra Pegu, Yi Zhang, Darcy R. Harris, Adam S. Olia, Chloe Adrienna Talana, Eun Sung Yang, Man Chen, Misook Choe, Wei Shi, I-Ting Teng, Adrian Creanga, Claudia Jenkins, Kwanyee Leung, Tracy Liu, Erik-Stephane D. Stancofski, Tyler Stephens, Baoshan Zhang, Yaroslav Tsybovsky, Barney S. Graham, John R. Mascola, Nancy J. Sullivan, Peter D. Kwong

**Affiliations:** ^1^ Vaccine Research Center, National Institute of Allergy and Infectious Diseases, National Institutes of Health, Bethesda, MD 20892, USA.; ^2^ Electron Microscopy Laboratory, Cancer Research Technology Program, Leidos Biomedical Research, Frederick National Laboratory for Cancer Research, Frederick, MD 21702, USA.

## Abstract

The rapid spread of the severe acute respiratory syndrome coronavirus 2 (SARS-CoV-2) B.1.1.529 (Omicron) variant and its resistance to neutralization by vaccinee and convalescent sera are driving a search for monoclonal antibodies with potent neutralization. To provide insight into effective neutralization, we determined cryo–electron microscopy structures and evaluated receptor binding domain (RBD) antibodies for their ability to bind and neutralize B.1.1.529. Mutations altered 16% of the B.1.1.529 RBD surface, clustered on an RBD ridge overlapping the angiotensin-converting enzyme 2 (ACE2)–binding surface and reduced binding of most antibodies. Substantial inhibitory activity was retained by select monoclonal antibodies—including A23-58.1, B1-182.1, COV2-2196, S2E12, A19-46.1, S309, and LY-CoV1404—that accommodated these changes and neutralized B.1.1.529. We identified combinations of antibodies with synergistic neutralization. The analysis revealed structural mechanisms for maintenance of potent neutralization against emerging variants.

## Acting against Omicron

The rapid spread of the Omicron variant of the severe acute respiratory syndrome coronavirus 2 (SARS-CoV-2) virus was partly due to its ability to evade antibodies elicited against the viral spike protein of early variants. Zhou *et al*. identified antibodies that maintain the ability to neutralize the Omicron variant and use functional and structural studies to determine how they recognize the Omicron spike protein. Their studies identify antibody combinations with therapeutic potential and could inform the development of vaccines. —VV

## Abstract (structured)

**INTRODUCTION:**

The emergence and rapid spread of the B.1.1.529 (Omicron) variant of concern (VOC) with 37 mutations in the spike protein has raised alarm. Especially troublesome are the 15–amino acid substitutions in the receptor binding domain (RBD) because RBD-directed antibodies have been the only antibodies found to retain sufficient potency against other variants. To identify antibodies that effectively neutralize B.1.1.529, we evaluated RBD-directed antibodies for their ability to bind and neutralize B.1.1.529 and determined their modes of recognition using functional assays and cryo–electron microscopy (cryo-EM) structures.

**RATIONALE:**

The severe acute respiratory syndrome coronavirus 2 (SARS-CoV-2) B.1.1.529 VOC is substantially resistant to neutralization by most monoclonal antibodies and by vaccinee and convalescent sera. Identifying monoclonal antibodies that retain neutralization potency against this variant and understanding their structural mechanism of recognition should inform the development of vaccines and antibody therapeutics that maintain effectiveness.

**RESULTS:**

The cryo-EM structure of the B.1.1.529 spike in its prefusion conformation revealed a single RBD-up conformation, with RBD substitutions localized to the outer surface of the spike. Despite this localization, RBD substitutions directly contacted or bordered epitopes of all previously identified RBD-directed neutralizing antibodies. Our studies revealed antibodies A23-58.1, B1-182.1, COV2-2196, S2E12, A19-46.1, S309, and LY-CoV1404 to nevertheless maintain substantial neutralization against this emerging variant. To provide structural and functional explanations, we determined cryo-EM structures of antibody-spike complexes and used virus particles representing each of the 15 single-amino-acid substitutions in RBD to delineate their functional impact. For class I and II antibodies that compete with angiotensin-converting enzyme 2 (ACE2) for binding to spike, such as VH1-58–derived antibodies B1-182.1 and S2E12, these analyses revealed potent neutralization to require smaller antibody side chains that accommodate the S477N mutation. For others, such as LY-CoV555 and A19-46.1, the epitopes of these antibodies bordered multiple RBD substitutions. Both E484A or Q493R greatly reduced binding for LY-CoV555, whereas for A19-46.1, these substitutions were generally tolerated, with the cryo-EM structure of A19-46.1 and spike revealing a two-RBD-up conformation and A19-46.1 binding only to up RBDs. For class III and IV antibodies that bind outside of the ACE2-binding surface—such as A19-61.1, COV2-2130, S309, and LY-CoV1404—individual B.1.1.529 substitutions were generally tolerated. However, A19-61.1 neutralization was eliminated by G446S; COV2-2130 showed substantially lower neutralization of B.1.1.529, but no single mutation exhibited a substantial impact; S309 retained potency against B.1.1.529, although not against the BA.2 sublineage variant (half-maximal inhibitory concentration reduced to 1374 ng/ml); and LY-CoV1404 retained potent neutralization (5.1 and 0.6 ng/ml against BA.1 and BA.2 sublineages, respectively). Last, we assessed combinations of monoclonal antibodies and found several, including the combination of B1-182.1 and A19-46.1, to show neutralization synergy. The structure of the ternary complex of B.1.1.529 spike with these two antibodies suggested the induction of the preferred up-RBD binding conformation by B1-182.1 to facilitate cooperative binding by A19-46.1 as the basis for their synergy.

**CONCLUSION:**

Although Omicron mutations cluster, they nonetheless affect virtually all known RBD-directed neutralizing antibodies. Our study reveals the structural basis by which select RBD-directed antibodies such as S2E12 and LY-CoV1404 retain potent neutralization of B.1.1.529. We further identified antibody combinations that can be used for treatment and have demonstrated how these combinations overcome extensive spike mutations.

## Teaser

Structural studies reveal how certain antibodies retain potent neutralization of the Omicron variant.

## Graphical abstract

**Figure:**
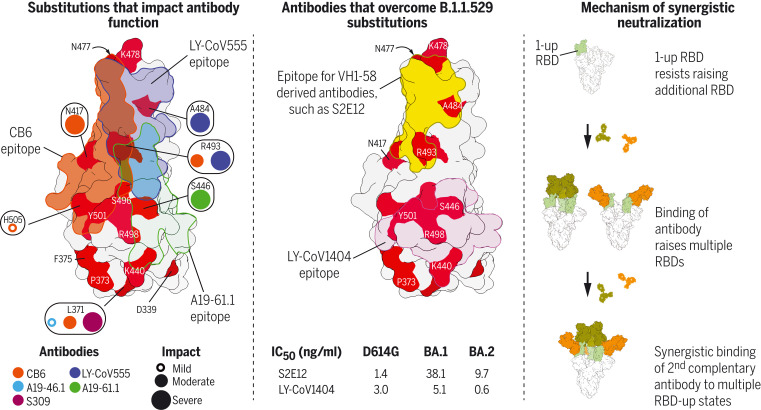
Structural basis for potent neutralization of B.1.1.529 by monoclonal antibodies and their combination. The RBD molecular surface is shown, colored by B.1.1.529 substitutions in red. (Left) Example epitopes are highlighted, along with the impact of each amino acid substitution. (Middle) Epitopes are shown for antibodies S2E12 and LY-CoV1404, which retain potent neutralization of B.1.1.529. (Right) Antibody combinations with complementary recognition modes can bind cooperatively to mediate synergistic neutralization.

Since first appearing in late 2019 (*1*), severe acute respiratory syndrome coronavirus 2 (SARS-CoV-2) has infected more than 490 million people and resulted in more than 5.9 million deaths (*2*). The appearance and rapid spread of the B.1.1.529 (Omicron; BA.1) variant (*3*, *4*)—with 34 amino acid substitutions, deletions, and insertions in the spike protein, which is three times higher than found in prior variants—has raised alarm. Although extremely broad antibodies such as S2P6 (*5*) that neutralize diverse β-coronaviruses, including SARS-CoV-2, are likely to be unencumbered by B.1.1.529 mutations, these broad antibodies neutralize in the microgram per milliliter range, whereas current therapeutic antibodies generally neutralize in the 1 to 50 nanogram per milliliter range for the ancestral D614G virus. (Single-letter abbreviations for the amino acid residues are as follows: A, Ala; C, Cys; D, Asp; E, Glu; F, Phe; G, Gly; H, His; I, Ile; K, Lys; L, Leu; M, Met; N, Asn; P, Pro; Q, Gln; R, Arg; S, Ser; T, Thr; V, Val; W, Trp; and Y, Tyr. In the mutants, other amino acids were substituted at certain locations; for example, D614G indicates that aspartate at position 614 was replaced by glycine.)

## Cryo-EM structure of B.1.1.529 (Omicron) spike

**Fig. 1.**
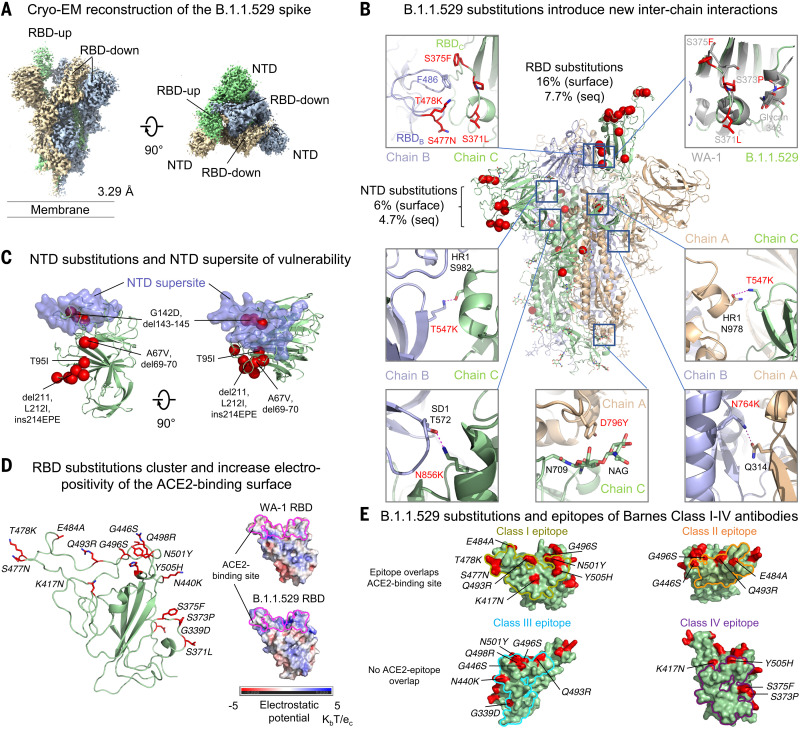
Cryo-EM structure of the SARS-CoV-2 B.1.1.529 (Omicron) spike. (**A**) Cryo-EM map of the SARS-CoV-2 B.1.1.529 spike. Reconstruction density map at 3.29 Å resolution is shown with side and top views. Protomers are colored light green, wheat, and light blue. The contour level of cryo-EM map is 4.0σ. (**B**) B.1.1.529 amino acid substitutions introduced interprotomer interactions. Substitutions in one of the protomers are shown as red spheres. Examples of interprotomer interactions introduced by B.1.1.529 substitutions are highlighted in the box with zoom-in views to the side. Amino acid substitutions are described as a percentage of the domain surface (surface) or as a percentage of the sequence (seq). (**C**) The NTD supersite of vulnerability is shown in semitransparent surface along with a green backbone ribbon. Amino acid substitutions, deletions, and insertions are in red. (**D**) The 15 amino acid substitutions, clustered on the rim of RBD, changed 16% of the RBD surface area (left) and increased electropositivity of the ACE2-binding site (right). Amino acid substitutions are shown as red sticks. The ACE2-binding site on the electrostatic potential surface are marked as magenta lines. (**E**) Mapping B.1.1.529 RBD substitutions on the epitopes of Barnes class I to IV antibodies. The locations of the substitutions are shown in red on the surface. Those that may potentially affect the activity of antibodies in each class are labeled with their residue numbers. Class I footprint is defined by epitopes of CB6 and B1-182.1; class II footprint is defined by epitopes of A19-46.1 and LY-CoV555; class III footprint is defined by epitopes of A19-61.1, COV2-2130, LY-CoV1404 and S309; and class IV footprint is defined by epitopes of DH1047 and S304. Class I and II antibodies primarily target the ACE2 binding site, whereas the epitopes of class III and IV antibodies do not. Class II and III epitopes allow binding to WA-1 when RBD is in the up or down conformation, although the distinction between class I and II is more fluid, particularly with new variants that alter the accessibility of epitopes relative to WA-1. In addition, some antibodies, such as A19-46.1, can bind fully up intermediate states between up and down but cannot bind the fully down state. We therefore classified primarily by binding region.

To provide insight into the impact of B.1.1.529 mutations on spike, we expressed and produced the two proline-stabilized (S2P) (*6*) B.1.1.529 spike and collected single-particle cryo–electron microscopy (cryo-EM) data that resulted in a structure of the trimeric ectodomain at 3.29 Å resolution (Fig. 1, fig. S1, and table S1). Like other D614G-containing variants, the most prevalent spike conformation comprised the single–receptor binding domain (RBD)–up conformation (*7*). B.1.1.529 mutations present in the spike gene resulted in three deletions of two, three, and one amino acids, a single insertion of three amino acids, and 30 amino acid substitutions in the spike ectodomain (fig. S2A). As expected from the ~3% variation in sequence, the B.1.1.529 spike structure was extremely similar to the WA-1 spike structure, with an overall Cα-backbone root mean square deviation (RMSD) of 1.8 Å (0.5 Å for the S2 region); however, we did observe minor conformational changes in a few places. For example, the RBD S371L/S373P/S375F substitutions changed the conformation of their residing loop so that F375 in the RBD-up protomer interacted with F486 in the neighboring RBD-down protomer and locked this RBD in down position (Fig. 1B). Moreover, the S373P substitution in the next RBD-down protomer increased contact surface with the neighboring RBD in down position, potentially latching itself in the down position (fig. S1G). All of these S371L/S373P/S375F substitution–mediated interactions help to stabilize the single-RBD-up conformation. Amino acid changes were denser in the N-terminal domain (NTD) and RBD, where most neutralizing antibodies bind, although RMSDs remained low (0.6 and 1.2 Å for NTD and RBD, respectively). About half the B.1.1.529 alterations in sequence outside the NTD and RBD involved new interactions, both hydrophobic, such as Y796 with the glycan on N709, and electrostatic, such as K547 and K856 interacting respectively with residues in heptad repeat 1 (HR1) in S2 and subdomain-1 (SD1) in S1 on neighboring protomers (Fig. 1B, fig. S2A, and table S2). Despite these newly introduced interactions, differential scanning calorimetry indicated that the B.1.1.529 spike had folding energy similar to that of the original WA-1 strain (fig. S2B).

NTD changes altered ~6% of the solvent-accessible surface on this domain, and several were located directly on or proximal to the NTD-supersite of vulnerability (*8*), where prior variants had mutations that substantially reduced neutralization by NTD antibodies. Other NTD changes neighbored a pocket, proposed to be the site of bilirubin binding (*9*), which also binds antibody (Fig. 1C) (*10*).

RBD alterations changed ~16% of the solvent-accessible surface on this domain and were constrained to the outward-facing ridge of the domain (Fig. 1D), covering much of the surface of the trimeric spike apex (fig. S1F). Several amino acid changes involved basic substitutions, resulting in a substantial increase in RBD electropositivity (Fig. 1D). Overall, RBD changes affected binding surfaces for the angiotensin-converting enzyme 2 (ACE2) receptor (Fig. 1D) (*11*) as well as recognition sites for potently neutralizing antibodies (Fig. 1E) (*12*–*14*).

## Functional assessment of variant binding to ACE2

**Fig. 2.**
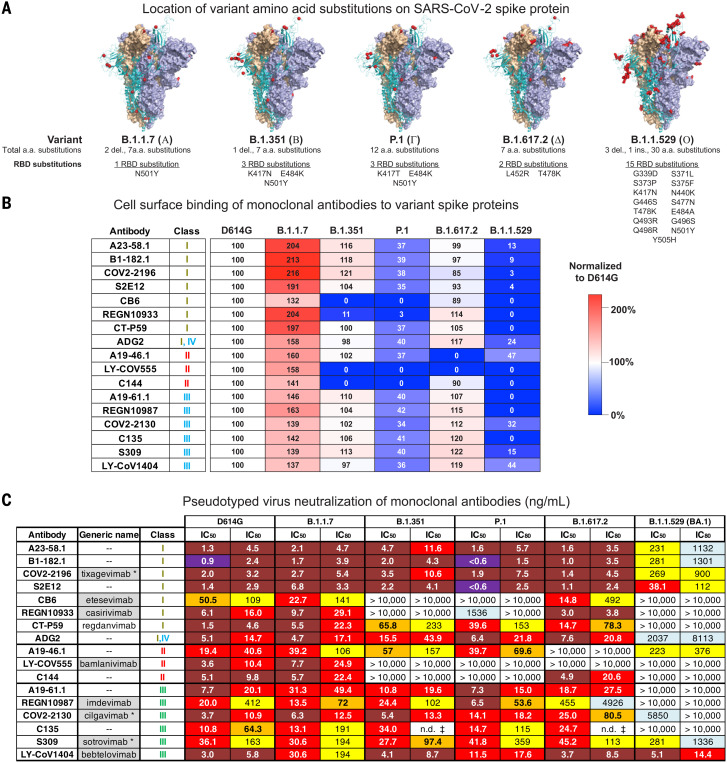
SARS-CoV-2 monoclonal antibody binding and neutralization. (**A**) Models of SARS-CoV-2 WA-1 spike protein (PDB: 6XM3) with the locations of substitutions present in variants indicated as red dots. Also indicated is the total number of amino acid substitutions and the number and locations of RBD substitutions in VOC spike proteins. (**B**) Full-length spike proteins from the indicated SARS-CoV-2 variants were expressed on the surface of transiently transfected 293T cells, and binding to indicated monoclonal antibodies was assessed by means of flow cytometry. Antibody mean fluorescence intensity (MFI) binding signal was adjusted according to spike protein expression level (fig. S4). Shown is the ratio of the adjusted antibody MFI binding to the indicated spike expressing cells to the adjusted MFI of the same antibody bound to D614G spike–expressing cells. The data are expressed as a percentage. Shown is a representative experiment (*n* = 2 replicates). (**C**) Lentiviruses pseudotyped with SARS-CoV-2 spike proteins from D614G, B.1.1.7, B.1.351, P.1, B.1.617.2, or B.1.1.529 (BA.1) were incubated with serial dilutions of the indicated antibodies, and IC_50_ and IC_80_ values were determined. S309 was tested on 293 flpin-TMPRSS2-ACE2 cells, whereas all the other antibodies were tested on 293T-ACE2 cells. Ranges are indicated with white (>10,000 ng/ml), light blue (>1000 to ≤10,000 ng/ml), yellow (>100 to ≤1000 ng/ml), orange (>50 to ≤100 ng/ml), red (>10 to ≤50 ng/ml), maroon (>1 to ≤10 ng/ml), and purple (≤1 ng/ml). n.d. ‡, not determined because of incomplete neutralization that plateaued at <80% (fig. S5B). Where available, generic names of antibodies under therapeutic investigation are shown. Gray shading indicates antibodies that previously or currently have received Emergency Use Authorization from the US Food and Drug Administration. Generic names with an asterisk indicate therapeutic antibody products with the same binding regions as those of the antibodies being tested but containing amino acid changes in their Fc domains.

When pathogens infect a new species, sustained transmission leads to adaptations that optimize replication, immune avoidance, and transmission. One hypothesis for the efficient species adaptation and transmission of SARS-CoV-2 in humans is that the virus spikes are evolving to optimize binding to the host receptor protein, ACE2. As a first test of this hypothesis, we used a flow cytometric assay to evaluate binding of human ACE2 to cells expressing variant spike proteins. We evaluated the binding of soluble dimeric ACE2 to B.1.1.7 (Alpha) (*15*), B.1.351 (Beta) (*16*), P.1 (Gamma) (*17*, *18*), or B.1.617.2 (Delta) (*19*) spikes compared with the ancestral D614G spike. The early B.1.1.7 variant contains an RBD substitution at N501Y (Fig. 2A), which increases RBD binding to ACE2 (*20*). Consistent with this, cell-surface ACE2 binding to B.1.1.7, which only contains an N501Y substitution in RBD, was 182% of D614G (fig. S3A). However, other N501Y-containing variants (B.1.351 and P.1) and B.1.617.2 (Fig. 2A), which lacks N501Y, did not show substantial increases in ACE2 binding signal (fig. S3A), a finding that is consistent with previous reports (*20*). Multiple groups have evaluated ACE2 binding to B.1.1.529 and found both increased or unchanged binding (*21*–*25*). In our cell binding assay, we found that ACE2 binding was 104% of the binding to D614G binding (fig. S3A).

Because cell-surface spike binding may be influenced by factors such as increased electropositivity of the RBD and by relative changes in the up/down state of RBD, we formally investigated the ACE2 binding affinity using surface plasmon resonance of soluble dimeric human ACE2 to S2P spike trimers generated from the ancestral WA-1 and six subsequent variants: D614G, B.1.351, P.1, B.1.617.2, B.1.1.7, and B.1.1.529. We observed that both WA-1 and D614G, which have identical RBD sequences, have similar apparent affinities (*K*_app_ = 1.1 and 0.73 nM, respectively) (fig. S3, B and C). The apparent affinity for variants was minimally changed (*K*_app_ = 0.59 to 3.8 nM), including for N501Y-containing variants (fig. S3, B and C). Given the minimal changes to affinity, our data suggests that spike variant evolution is not being driven by the optimization of ACE2 binding but is instead driven primarily by immune pressure.

## Variant binding and neutralization by individual monoclonal antibodies

To define the impact of SARS-CoV-2 variant amino acid changes on the binding and neutralization of monoclonal antibodies, we expressed and purified 17 highly potent antibodies targeting the spike RBD (*12*, *13*, *26*–*38*), including 13 antibodies currently under clinical investigation or approved for use under emergency use authorization (EUA) by the US Food and Drug Administration. All antibodies bound and neutralized B.1.1.7 comparable with the ancestral D614G and consistent with the single 501Y substitution being outside each antibody’s binding epitope (Fig. 2 and fig. S5A). Consistent with previous reports (*14*, *39*–*42*), two additional RBD substitutions in the RBD of B.1.351 and P.1 variants (Fig. 2A) led to substantially decreased binding and neutralization by the two class I antibodies CB6 and REGN10933 and the two class II antibodies LY-CoV555 and C144 (Fig. 2, B and C, and fig. S5A). In addition, whereas binding of CT-P59 to B.1.351 and P.1 variants was minimally changed (37 to 100%), neutralization was decreased 26- to 43-fold (Fig. 2, B and C). The remaining antibodies showed minimal binding changes and a <3.6-fold difference in neutralization half-maximal inhibitory concentration (IC_50_) (Fig. 2, B and C, and fig. S5A). An evaluation of the antibodies in our panel against B.1.617.2 revealed minimal changes in binding and neutralization for all antibodies except REGN10987, A19-46.1, and LY-CoV555 (Fig. 2, B and C). As previously reported (*14*, *39*–*42*), REGN10987 binds B.1.617.2 spike but has 22-fold less neutralization, and the binding and neutralizing activity of A19-46.1 and LY-CoV555 was eliminated (Fig. 2, B and C). These data are consistent with previous results that showed both A19-46.1 and LY-CoV555 were sensitive to the L452R mutations present in B.1.617.2 (*14*, *39*, *40*).

For B.1.1.529, all but three antibodies (A19-46.1, COV2-2130, and LY-CoV1404) showed binding less than 32% of D614G. Furthermore, although COV2-2196, S2E12, B1-182.1, and A23-58.1 use the same VH1-58 gene in their heavy chain and target a similar region on the RBD (the VH1-58 supersite), they showed differential binding to B.1.1.529 (3, 4, 9, and 13%, respectively) and B.1.617.2 (85, 93, 97, and 99%, respectively) (Fig. 2B). Even though the absolute differences in binding are minimal, the shared trend may be reflective of how the RBD tip T478K substitution found in B1.1.529 and B1.617.2 is accommodated by each of these antibodies. Taken together, cell surface binding suggests that whereas both A19-46.1 (47%) and LY-CoV1404 (44%) are likely to retain potent neutralizing activity against B.1.1.529, the remaining antibodies in our panel might show decreased neutralizing activity.

Using the same panel of monoclonal antibodies, we further assayed for each antibody’s capacity to neutralize the B.1.1.529 variant. VH1-58 supersite antibodies are a subset of class I antibodies that bind to the tip of RBD and have high neutralization activity against previous variants (*14*); despite this, their IC_50_s were 40- to 126-fold worse against B.1.1.529 relative to D614G (Fig. 2C). In addition, two other antibodies, CB6 (class I) and ADG2 (class I/IV), were severely affected (Fig. 2C). Among the class II antibodies (LY-CoV555, C144, and A19-46.1), neutralization by LY-CoV555 and C144 was completely abolished. By contrast, we found that the A19-46.1 neutralization IC_50_ was 223 ng/ml for B.1.1.529 versus 19.4 ng/ml for D614G (Fig. 2C) and was less than sixfold of the previously reported IC_50_ for WA-1 (39.8 ng/ml) (*14*). For class III antibodies, neutralization activity of A19-61.1, REGN10987, and C135 was completely abolished; CoV2-2130 decreased 1581-fold; and that of S309 decreased by approximately eightfold (Fig. 2C). In contrast to all the other antibodies, we found that the neutralization of LY-CoV1404 against B.1.1.529 was unchanged relative to D614G (Fig. 2C). Taken together, these data demonstrate that the mutations present in B.1.1.529 mediate resistance to a broad range of antibodies.

## Structural and functional basis of class I antibody neutralization, escape, and retained potency

**Fig. 3.**
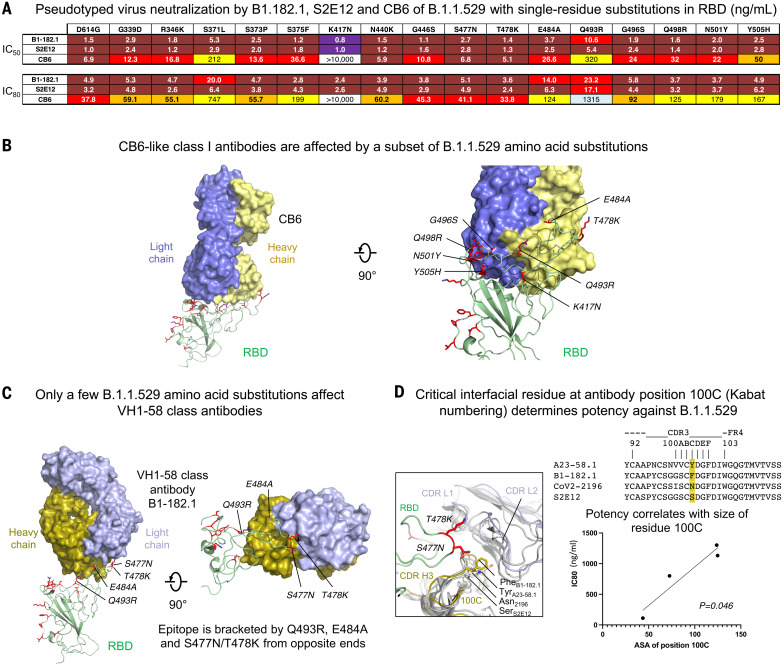
Functional and structural basis of class I antibody neutralization and mechanistic basis of retained potency against B.1.1.529 VOC. (**A**) Lentiviruses pseudotyped with SARS-CoV-2 spike proteins from D614G or D614G plus the indicated point substitutions found within the B.1.1.529 spike were incubated with serial dilutions of the indicated antibodies, and IC_50_ and IC_80_ values were determined on 293T-ACE2 cells. Ranges are indicated with white (>10,000 ng/ml), light blue (>1000 to ≤10,000 ng/ml), yellow (>100 to ≤1000 ng/ml), orange (>50 to ≤100 ng/ml), red (>10 to ≤50 ng/ml), maroon (>1 to ≤10 ng/ml), and purple (≤1 ng/ml). (**B**) Mapping of B.1.1.529 amino acid substitutions at the epitope of class I antibody CB6. RBD-bound CB6 was docked onto the B.1.1.529 spike structure. B.1.1.529 amino acid substitutions incompatible with CB6 binding were identified and labeled. The K417N substitution caused a clash in the center of the paratope. B.1.1.529 RBD is shown in green cartoon, with amino acid substitutions as red sticks. CB6 is shown in surface representation, with heavy and light chains in yellow and slate, respectively. (**C**) Docking of RBD-bound VH1-58–derived class I antibody B1-182.1 onto the B.1.1.529 spike structure identified four substitutions with potential steric hindrance. B1-182 is shown in surface representation, with heavy and light chains colored olive and light blue, respectively. B.1.1.529 amino acid substitutions that may affect binding of VH1-58 antibodies were labeled. (**D**) Structural basis for effective neutralization of the B.1.1.529 VOC by VH1-58–derived antibodies. Even though VH1-58 antibodies—such as the S2E12, COV2-2196, A23-58.1, and B1-182.1—share high-sequence homology (top right), their neutralization potency against B.1.1.529 varies. Structural analysis indicated that CDR H3 residue 100C, located at the interface formed between RBD and antibody heavy and light chains, may determine their potency against B.1.1.529 (left). Size of this residue correlated with neutralization potency with two-tailed *P* = 0.046 (bottom right).

To determine the functional basis of B.1.1.529 neutralization and escape for class I antibodies, we analyzed class I antibodies CB6, B1-182.1, and S2E12, which show differential B.1.1.529 neutralization (Fig. 2C). CB6 is a class I antibody that does not use the VH1-58 gene and whose epitope is partially overlapping with the VH1-58 supersite. We used virus particles containing single–amino acid substitutions representing each of 15 single–amino acid changes on the RBD of B.1.1.529. Whereas K417N completely abrogated neutralization of CB6, the presence of Y505H, S371L, or Q493R substitutions decreased neutralization from 7- to 46-fold (Fig. 3A). Taken together, this suggests that B.1.1.529 evades CB6-like antibodies through multiple substitutions. Docking of the RBD-bound CB6 onto the B.1.1.529 structure revealed several B.1.1.529 substitutions that may affect CB6 binding through a steric clash (Q493R) and removal of key contacts (K417N and Y505H), which is consistent with neutralization data (Fig. 3B). The VH1-58 supersite antibodies B1-182.1 and S2E12 have similar amino acid sequences to each other (*14*) but show an approximately sixfold difference in B.1.1.529 neutralization. These two antibodies remained highly potent for all virus particles with single RBD mutations (IC_50_ < 10.6 ng/ml), with the largest change for Q493R, which caused a 7- and 5.4-fold decrease of neutralization for B1-182.1 and S2E12, respectively (Fig. 3A). These small differences in neutralization from single mutations suggest that two or more combinations of mutations of B.1.1.529 are working in concert to mediate escape from VH1-58 supersite antibodies. Docking of the RBD-bound B1-182.1 onto the B.1.1.529 structure indicated that the epitopes of these antibodies were bounded by Q493R, S477N, T478K, and E484A, with R493 pressing on one side of the antibody and N477/K478 on the other side of the antibody at the heavy chain–light chain interface (Fig. 3C). N477/K478 positioned at the junction formed by complementarity-determining region (CDR) H3, CDR L1, and L2 and clashed slightly with a region centered at CDR H3 residue 100C (Kabat numbering) (Fig. 3D). Sequence alignment of CDR H3 of VH1-58–derived antibodies indicated that residue 100C varies in side chain size, from serine in S2E12 to tyrosine in A23-58.1. The size of 100C reversely correlated with neutralization potency IC_80_ (*P* = 0.046) (Figs. 2C and 3D), suggesting that VH1-58 antibodies could alleviate escape imposed by the B.1.1.529 mutations through reduced side chain size at position 100C to minimize clashes from N477/K478.

## Structural and functional basis of class II antibody neutralization, escape, and retained potency

**Fig. 4.**
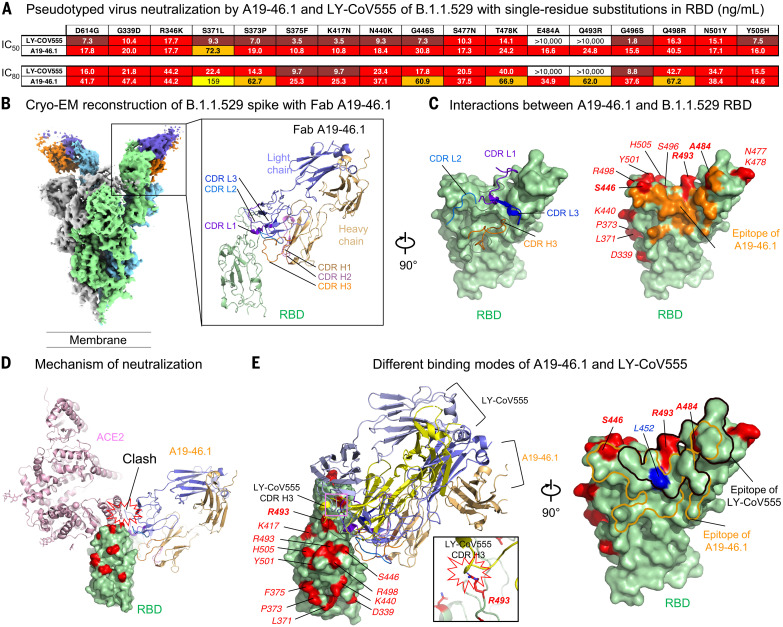
Functional and structural basis of class II antibody binding, neutralization, and escape. (**A**) Lentiviruses pseudotyped with SARS-CoV-2 spike proteins from D614G or D614G plus the indicated point substitutions found within the B.1.1.529 spike were incubated with serial dilutions of the indicated antibodies, and IC_50_ and IC_80_ values were determined on 293T-ACE2 cells. Ranges are indicated with white (>10,000 ng/ml), light blue (>1000 to ≤10,000 ng/ml), yellow (>100 to ≤1000 ng/ml), orange (>50 to ≤100 ng/ml), red (>10 to ≤50 ng/ml), maroon (>1 to ≤10 ng/ml), and purple (≤1 ng/ml). (**B**) Cryo-EM structure of class II antibody A19-46.1 Fab in complex with the B.1.1.529 spike. Overall density map is shown to the left, with protomers in light green, gray, and light cyan. Two A19-46.1 Fabs bound to the RBD in the up conformation are shown in orange and slate. Structure of the RBD and A19-46.1 after local focused refinement is shown to the right in cartoon representation. The heavy-chain CDRs are in brown, pink, and orange for CDR H1, CDR H2, and CDR H3, respectively. The light chain CDRs are in marine purple blue, marine blue, and blue for CDR L1, CDR L2, and CDR L3, respectively. The contour level of the cryo-EM map is 4.0σ. (**C**) Interaction between A19-46.1 and RBD. (Left) CDR H3 and all light-chain CDRs that are involved in binding of RBD. Epitope of A19-46.1 is shown in orange on the green B.1.1.529 RBD surface, with amino acid substitutions in red. (Right) S446, A484, and R493 are located at the edge of the epitope of Fab A19-46.1. RBD residues are labeled with italicized font. (**D**) Binding of A19-46.1 to RBD prevents binding of the ACE2 receptor. ACE2 and A19-46.1 are shown in cartoon representation. (**E**) Comparison of binding modes to RBD for antibody A19-46.1 and LY-CoV555. (Left and inset) Even though both antibodies target similar regions on RBD, different approaching angles caused a clash between LY-CoV555 CDR H3 and B.1.1.529 substitution R493. (Right) B.1.1.529 substitutions involved in binding of A19-46.1 are only at the edge of its epitope, whereas both R493 and A484 locate in the middle of LY-CoV555 epitope. L452R substitution that eliminates A19-46.1 and LY-CoV555 binding in other SARS-CoV-2 variants is in blue.

We next sought to determine the functional basis of B.1.1.529 neutralization and escape for two class II antibodies, LY-CoV555 (*31*) and A19-46.1 (*14*), which have B.1.1.529 IC_50_ of >10,000 and 223 ng/ml, respectively (Fig. 2C). Consistent with previous reports (*14*, *43*, *44*), either E484A or Q493R substitution results in complete loss of LY-CoV555 neutralization, whereas the same mutations did not affect A19-46.1 (Fig. 4A). For A19-46.1, no individual mutation reduced neutralization to the level noted in B.1.1.529 except S371L, which increased the IC_50_ to 72.3 ng/ml (Fig. 4A). In the context of B1.1.529, which contains the S371L/S373P/S375F alterations, the IC_50_ further increased to 223 ng/ml (Fig. 2C). One potential explanation for this further reduction of potency is that the mutation-introduced interaction between F375 and F486 (Fig. 1B) restricts the RBD-up conformation required for A19-46.1 binding.

To understand the structural basis of A19-46.1 neutralization of B.1.1.529, we obtained a cryo-EM structure of the B.1.1.529 spike in complex with Fab A19-46.1 at 3.86 Å resolution (Fig. 4B, fig. S6, and table S1). Two Fabs bound to the RBDs in up-conformation in each spike, with the third RBD in down position. Docking Fab A19-46.1 onto the RBD in down conformation revealed a clash with the NTD of the neighboring protomer, suggesting that A19-46.1 binding requires the RBD-up conformation. Focused local refinement of the antibody-RBD region resolved the antibody-RBD interface (Fig. 4B, right). Consistent with previous mapping and negative stain EM data, A19-46.1 binds to a region on RBD generally targeted by class II antibodies with an angle ~45° toward the viral membrane. Binding involves all light chain CDRs and only CDR H3 of the heavy chain and buries a total of 805 Å^2^ interface area from the antibody (Fig. 4C, left). With the light chain latching to the outer rim of the RBD and providing about 70% of the binding surface, A19-46.1 uses its 17-residue-long CDR H3 to form parallel strand interactions with RBD residues 345 to 350 (Fig. 4B, right). Docking RBD-bound ACE2 to the A19-46.1–RBD complex indicated that the bound antibody sterically clashes with ACE2 (Fig. 4D), providing the structural basis for its neutralization of B.1.1.529.

The 686 Å^2^ epitope of A19-46.1 is located within an RBD region that is not mutated in B.1.1.529. Three of the 15 amino acid changes on the RBD, S446, A484, and R493 are positioned at the edge of epitope, with their side chains contributing 8% of the binding surface. LY-CoV555, which targets the same region as that of a class II antibody, completely lost activity against B.1.1529. Superimposing the LY-CoV555-RBD complex onto the B.1.1.529 RBD showed that although LY-CoV555 has an angle of approach similar to that of A19-46.1 (Fig. 4D), its epitope shifts up to the ridge of the RBD and includes the B.1.1.529 alterations A484 and R493 (Fig. 4D). R493 causes steric clash with the CDR H3 of LY-CoV555, explaining the escape of B.1.1.529 from LY-CoV555 neutralization. Overall, the location of the epitope and the angle of approach allow A19-46.1 to effectively neutralize B.1.1.529.

## Structural and functional basis of class III antibody neutralization, escape, and retained potency

**Fig. 5.**
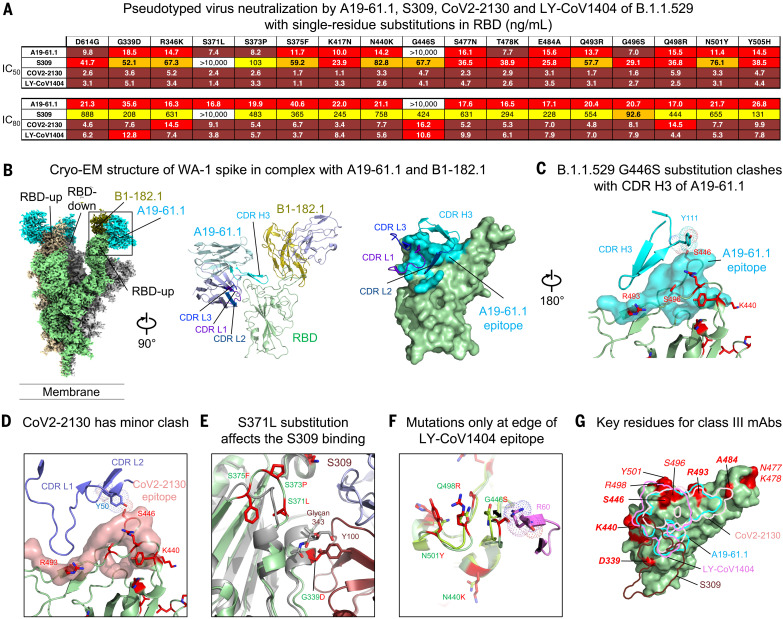
Functional and structural basis of class III antibody binding, neutralization, and retained potency against the B.1.1.529 VOC. (**A**) Lentiviruses pseudotyped with SARS-CoV-2 spike proteins from D614G or D614G plus the indicated point substitutions found within the B.1.1.529 spike were incubated with serial dilutions of the indicated antibodies, and IC_50_ and IC_80_ values were determined. A19-61.1 and LY-COV1404 were assayed on 293T-ACE2 cells, whereas S309 and CoV2-2130 were tested on 293 flpin-TMPRSS2-ACE2 cells. Ranges are indicated with white (>10,000 ng/ml), light blue (>1000 to ≤10,000 ng/ml), yellow (>100 to ≤1000 ng/ml), orange (>50 to ≤100 ng/ml), red (>10 to ≤50 ng/ml), maroon (>1 to ≤10 ng/ml), and purple (≤1 ng/ml). (**B**) Cryo-EM structure of SARS-CoV-2 WA-1 spike in complex with class I antibody B1-182.1 and class III antibody A19-61.1 at 2.83 Å resolution. Overall density map is shown, with protomers in light green, gray, and wheat. Two RBDs are in the up conformation, with each binding both Fabs, and one RBD is in the down position, with A19-61.1 bound. (Left) RBD. B1-182.1 and A19-61.1 are in olive and cyan, respectively. Structure of the RBD with both Fabs bound after local focused refinement is shown to the right in cartoon representation. (Middle) RBD is shown in green cartoon, and antibody light chains are in light blue. (Right) Epitope of A19-61.1 is shown as cyan surface on RBD, with interacting CDRs labeled. The contour level of cryo-EM map is 5.2σ. (**C**) Structural basis of B.1.1.529 resistance to A19-61.1. Mapping of the A19-61.1 epitope onto the B.1.1.529 RBD indicated that G446S clashed with CDR H3 of A19-61.1. RBD is shown in green cartoon, with amino acid substitutions as red sticks, and epitope of A19-61.1 is the cyan surface. (**D**) Structural basis of CoV2-2130 neutralization of the B.1.1.529 VOC. Docking of the CoV2-2130 onto the B.1.1.529 RBD showed that Y50 in CDR L2 posed a minor clash with S446. RBD is shown in green cartoon, with amino acid substitutions as red sticks, and epitope of CoV2-2130 is the pink surface. (**E**) Structural basis of S309 neutralization of the B.1.1.529 VOC. Docked complex of S309 and B.1.1.529 RBD showed that the S371L/S373P/S375F Loop changed conformation, and the S371L substitution is adjacent to the S309 epitope, whereas the G339D substitution is located inside the epitope. D339 side-chain clashes with CDR H3 Y100. B.1.1.529 RBD is shown in green cartoon, with amino acid substitutions as red sticks, and WA-1 RBD is shown in gray cartoon. (**F**) Structural basis of LY-CoV1404 neutralization of the B.1.1.529 VOC. Docking of the LY-CoV1404 onto the B.1.1.529 RBD identified four amino acid substitutions in the epitope, with G446S causing a potential clash with CDR H2 R60. However, comparison of both LY-CoV1404–bound and –nonbound B.1.1.529 RBD indicated that the S446 loop has the flexibility to allow LY-CoV1404 binding. B.1.1.529 residues at LY-CoV1404 epitope are shown as red sticks, with corresponding WA-1 residues as green sticks. CDR H3 is shown in cartoon representation and colored magenta. (**G**) Overlay of epitope footprints of class III antibodies onto the B.1.1.529 RBD. Locations of amino acid substitutions in B.1.1.529 RBD are in red on the green surface.

To evaluate the functional basis of B.1.1.529 neutralization and escape for class III antibodies and to understand how potent neutralization might be retained, we investigated a panel of class III antibodies with differential potency, including A19-61.1, COV2-2130, S309, and LY-CoV1404 (Fig. 5A). Assessment of the impact of each of the 15 mutations in the RBD revealed that the G446S amino acid change results in a complete loss in activity for A19-61.1 (Fig. 5A), which is consistent with the complete loss of function of this antibody against B.1.1.529 and previous reports that suggested G446V might affect function (*14*). For S309, S373P resulted in a small change in neutralization (Fig. 5A). Unexpectedly, although S309 retains moderate neutralizing activity against B.1.1.529, we found that the single S371L amino acid change leads to a loss in S309 neutralization (Fig. 5A). This suggests that combinations of S371L with other B.1.1.529 mutations result in structural changes in spike that allow S309 to partially overcome the S371L change. None of the single–amino acid changes evaluated resulted in markedly different neutralization by COV2-2130 (Fig. 5A), suggesting that combinations of amino acid substitutions act in concert to decrease neutralization potency against B.1.1.529. Last, consistent with the overall high potency of LY-CoV1404 against all tested variants of concern (VOCs), we did not identify an amino acid change that affected its function.

To understand the structural basis of class III antibody neutralization and viral escape, we determined the cryo-EM structure of WA-1 S2P in complex with Fab A19-61.1 (and Fab B1-182.1 to aid EM resolution of local refinement) at 2.83 Å resolution (Fig. 5B, fig. S8, and table S1). The structure revealed that two RBDs were in the up-conformation with both antibodies bound, and the third RBD was in the down-position with only A19-61.1 bound, indicating that A19-61.1 could recognize RBD in both up and down conformation (Fig. 5B). Local refinement of the RBD-Fab A19-61.1 region showed that A19-61.1 targets the class III epitope with interactions provided by the 18-residue-long CDR H3 from the heavy chain and all CDRs from the light chain (Fig. 5B). Docking the A19-61.1 structure to the B.1.1.529 spike structure indicated that B.1.1.529 mutations S446, R493, and S496 might interfere with A19-61.1. Analysis of the side-chain interactions identified a clash between Y111 in CDR H3 and S446 in the RBD that could not be resolved with loop flexibility (Fig. 5C), explaining the loss of A19-61.1 neutralization against G446S-containing SARS-CoV-2 variants.

Neutralization assays indicated that among the class III antibodies, COV2-2130, S309, and LY-CoV1404 showed variable neutralization potency against B.1.1.529. Docking indicated that CoV2-2130 targets an epitope very similar to A19-61.1, with interactions mainly mediated by its CDR L1 and L2 and avoiding close contact with R493 and S496. However, the OH group of Y50 in CDR L2 showed a minor clash with S446 in RBD, which explains the structural basis for the partial conservation of neutralization by CoV2-2130 (Fig. 5D). Antibody S309 showed higher potency against B.1.1.529 than CoV2-2130. In a docked complex, the G339D mutation is located inside the epitope and clashes with CDR H3 Y100; however, the void space between S309 and RBD might accommodate an alternate tyrosine rotamer. The S371L/S373P/S375F mutations changed the conformation of their residing loop and may push the glycan on N343 toward S309 to reduce binding (Fig. 5E). LY-CoV1404 was not affected by B.1.1.529 mutations. Docking of the LY-CoV1404 onto the B.1.1.529 RBD identified four amino acid substitutions located at the edge of its epitope. Three of the residues—K440, R498, and Y501—only make limited side-chain interactions with LY-CoV1404. The fourth residue, G446S, caused a potential clash with CDR H2 R60. However, comparison of LY-CoV1404–bound and –nonbound RBD indicated that the loop containing S446 had conformational flexibility that could allow LY-CoV1404 binding (Fig. 5F). Overall, the epitopes to class III antibodies were mainly located on mutation-free RDB surfaces with edges contacting a few B.1.1.529 alterations (Fig. 5G). LY-CoV1404 retained high potency by accommodating all four B.1.1.529 alterations at the edge of its epitope by exploiting loop mobility or by minimizing side-chain interactions.

## Synergistic neutralization by the combination of B1-182-1 and A19-46.1

**Fig. 6.**
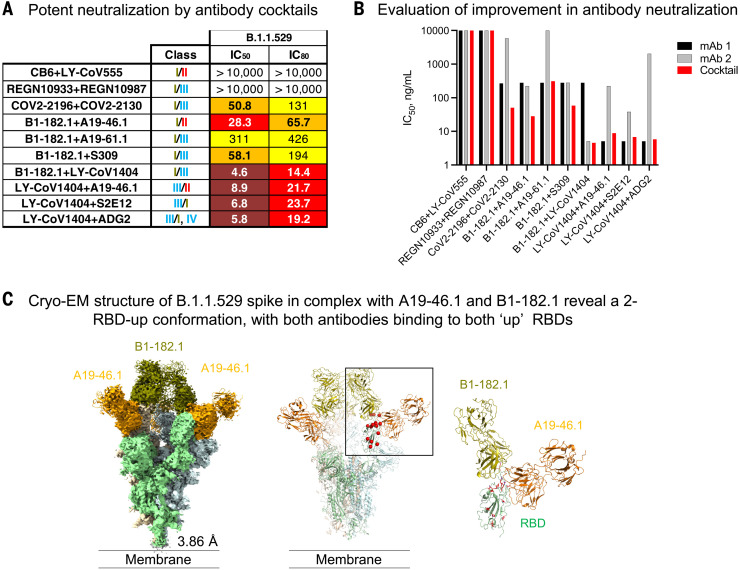
Potent neutralization of SARS-CoV-2 B.1.1.529 using combinations of antibodies. (**A**) Lentiviruses pseudotyped with SARS-CoV-2 B.1.1.529 spike were incubated with serial dilutions of the indicated combination of antibodies, and IC_50_ and IC_80_ values were determined. Ranges are indicated with white (>10,000 ng/ml), light blue (>1000 to ≤10,000 ng/ml), yellow (>100 to ≤1000 ng/ml), orange (>50 to ≤100 ng/ml), red (>10 to ≤50 ng/ml), maroon (>1 to ≤10 ng/ml), and purple (≤1 ng/ml). (**B**) Neutralization IC_50_ (ng/ml) values for each of the indicated cocktail (*x* axis) or its component antibodies. The IC_50_ for first antibody is listed as mAb1 (black), and the second antibody is listed as mAb2 (gray) or cocktail (red). (**C**) Cryo-EM structure of B.1.1.529 spike in complex with antibodies A19-46.1 and B-182.1 at 3.86 Å resolution. (Left) Overall density map, with protomers colored light green, wheat, and light cyan. (Middle) All RBD are in up conformation, with both Fabs bound. (Right) Binding of one Fab (such as B1-182.1) induces RBD into the up conformation and potentially facilitates binding of the other Fab (such as A19-46.1), which only recognizes the up conformation of RBD. A19-46.1 and B-182.1 are in orange and olive, respectively. The contour level of cryo-EM map is 6.5σ.

We previously reported that the combination of B1-182.1 and either A19-46.1 or A19-61.1 mitigated mutational escape in an in vitro virus escape assay (*14*), which suggests the possibility of synergistic neutralization. To look for other synergistic combinations, we determined the neutralization of B.1.1.529 pseudotyped viruses through clinically used cocktails or various combinations of B1-182.1, A19-46.1, A19-61.1, LY-CoV1404, ADG2, and S309. Of the 10 combinations evaluated, only COV2-2196+COV2-2130, B1-182.1+A19-46.1, and B1-182.1+S309 neutralized B.1.1.529 with an appreciably improved potency (IC_50_ of 50.8, 28.3, and 58.1 ng/ml, respectively) over the individual component antibodies (Fig. 6, A and B). Each of these included a VH-158 supersite antibody and showed a five- to 115-fold improvement over the component antibodies (Fig. 6B), suggesting an effect that is more than an additive for the specific combination against B.1.1.529.

To understand the structural basis of the improved neutralization by the cocktail of B1-182.1 and A19-46.1, we determined the cryo-EM structure of the B.1.1.529 S2P spike in complex with Fabs of B1-182.1 and A19-46.1 at 3.86 Å resolution (Fig. 6C, fig. S9, and table S1). Three-dimensional reconstruction revealed that the combination of these two antibodies induced the spike to a three-RBD-up conformation, with both Fabs bound to each RBD (although Fabs on one of the RBDs were lower in occupancy). The spike had a 1.6 Å RMSD relative to the three-RBD-up WA-1 structure [Protein Data Bank (PDB) ID: 7KMS]. Overall, the structure showed that these two antibodies were capable of simultaneously recognizing the same RBD, and the combination increased the overall stoichiometry compared with two Fabs per trimer observed in the S2P-A19-46.1 structure described above. Of all the antibodies tested, all VH1-58–derived antibodies retained reasonable levels of neutralization against B.1.1.529, whereas some members of other antibody classes suffered complete loss of activity. VH1-58 antibodies have few alterations in their B1.1.529 epitopes and can evolve means to alleviate the impact. We propose a model for the B1-182.1 and A19-46.1 cocktail, in which binding of the first antibody induces the spike into an RBD-up conformation and thereby facilitates the binding of the second antibody that prefers the up conformation. This would kinetically favor an all–RBD-up state by “trapping” RBD in the up position and could lead to a synergistic increase in neutralization potency compared with that of the individual antibodies. This model of synergistic neutralization of Omicron-related viruses and other combinations of antibodies that target the RBD will need further investigation.

## Discussion

SARS-CoV2 VOCs provide a window into the coevolution of key host-pathogen interactions between the viral spike, human ACE2 receptor, and humoral immune responses. The RBD is a major target for neutralizing antibodies in both convalescents and vaccinees. An understanding of how RBD mutations evolve may guide the development and maintenance of effective antibody therapeutics and vaccines.

We found that in the context of trimeric spike proteins, variant amino acid changes did not provide a biologically meaningful alteration in affinity to ACE2. When binding trimeric spike protein to immobilized ACE2, our analysis showed that the apparent affinity of B.1.1.529 to ACE2 only changed approximately threefold compared with that of WA-1 (*K*_Dapp_ = 3.8 nM versus 1.1 nM for WA-1), which is consistent with the 1.4-fold observed by Mannar *et al*. (2.1 nM versus 3.0 nM) (*21*). When tested in the context of RBD, affinity to immobilized ACE2 also showed less than twofold variation between B.1.1.529 and WA-1 (*22*–*25*). This suggests that there is either no further fitness benefit to be gained by improving affinity, that affinity improving changes are being used to compensate for mutations that are deleterious for ACE2 binding but allow immune escape, or both.

Our findings for the class I VH1-58 supersite showed that B.1.1.529 acquires a series of mutations that are not individually deleterious yet bracket the antibody and reduce its potency. VH1-58 antibodies can alleviate the impact by reducing the size of CDR H3 residue 100C to avoid clashes from B.1.1.529 mutations. Because VH1-58 supersite are among the most potent and broadly neutralizing antibodies to SARS-CoV-2 (*14*, *30*, *34*, *45*), our findings point the way toward structure-based designs of existing antibodies to mitigate against amino acid changes at these positions.

For the class II antibody A19-46.1, its preference to RBD in the up-conformation is different from LY-CoV555, which recognizes both RBD-up and -down conformation. The angle of approach and a long-CDR H3 allow A19-46.1 to target the mutation-free face on RBD and minimize contact with mutations on the RBD ridge of B.1.1.529. Comparing the effect of S371L on neutralization by A19-46.1 and LY-CoV555 (Fig. 4A) suggested that L371 (and potentially P373/F375) is critical for controlling the RBD-up or -down conformation in B.1.1.529. This concept is supported by the finding that combination with a class I antibody (such as B1-182.1) synergistically enhances A19-46.1 neutralization (Fig. 6A).

For class III antibodies, only one prototype antibody showed complete loss of B.1.1.529 neutralization. We determined that viral escape was mediated by the G446S amino acid change. This result indicates that potent class III antibodies might be induced through structure-based vaccine designs that mask residue 446 in RBD. Additionally, the existence of G446S-sensitive and -resistant antibodies with substantial epitope overlap suggest that spikes with the G446S substitution can be used to evaluate the quality of class III immune response in serum-based epitope mapping assays (*46*, *47*). In addition, we found that although S309 is severely affected by the S371L mutation alone, it is rescued by compensating mutations in B.1.1.529. Similar but less severe results were recently reported for Brii-198 (*48*, *49*). Taken together, this suggests that there may be a fitness advantage to the virus to maintain a surface that is compatible with S309 binding.

Our analysis of antibodies of clinical importance is consistent with previous reports (*37*, *50*–*52*) and showed that S309 and COV2-2196 neutralized B.1.1.529 to similar degrees. We report that unlike other antibodies, the highly potent LY-CoV1404 does not lose neutralization potency against B.1.1.529. In addition, most antibodies in our panel neutralized the recently described BA.2 Omicron variant (*53*) with similar potency (fig. S10, A and B). The exceptions were with the class III antibodies A19-61.1 and COV2-2130—which fully recovered their neutralization potency, potentially because of the absence of the G446S mutation in BA.2—and S309, which lost more than fivefold activity (fig. S10B).

We identified combinations of antibodies that show more than additive increases in neutralization against B.1.1.529—including COV2-2196+COV2-2130, B1-182.1+A19-46.1, and B1-182.1+S309—and all but the B1-182.1+S309 also show synergy against BA.2 (fig. S10C). Each pair contains a VH1-58 supersite antibody that only binds RBD in the up position and have been shown to be able to bind to all three RBD-up protomers (*14*). We speculate that antibodies that are not affected by S371L, such as VH1-58 mAbs, induce and stabilize the three–RBD-up conformation. This allows antibodies that prefer RBD-up conformation—and would otherwise be unable to break the RBD-down locking conformation imposed by the mutations at 371, 373, and 375—to more efficiently bind. This identification of SARS-CoV-2 monoclonal antibodies that function cooperatively is similar to that seen previously for other viruses (*54*) and supports the concept of using combinations to both enhance potency and mitigate the risk of escape.

## Materials and methods

### Expression and purification of proteins

Soluble 2P-stabilized SARS-CoV-2 spike proteins were expressed by transient transfection (*6*, *55*). Briefly, plasmid was transfected using Expifectamine (Gibco, #A14525) into Expi293F cells (Gibco, #A14527) and the cultures enhanced 16 to 24 hours post-transfection. Following 4 to 5 days incubations at 120 rotations per minute (rpm), 37°C, 9% CO2, supernatant was harvested, clarified via centrifugation, and buffer exchanged into 1X PBS. Protein of interests were then isolated by affinity chromatography using Ni-NTA resin (Roche, #589380101) followed by size exclusion chromatography on a Superose 6 increase 10/300 column (GE healthcare, #29091596)).

Expression and purification of biotinylated S2P used in binding studies were produced by an in-column biotinylation method as previously described (*55*). Using full-length SARS-Cov2 S and human ACE2 cDNA ORF clone vector (Sino Biological, #HG10108-CH) as the template to the ACE2 dimer proteins. The ACE2 PCR fragment (1~740aa) was digested with Xbal (New England Biolabs, #R3136S) and BamHI (New England Biolabs, #R0145S) and cloned into the VRC8400 with Avi-HRV3C-single chain-human Fc-his (6x) tag on the C-terminal. All constructs were confirmed by sequencing. Proteins were expressed in Expi293 cells by transfection with expression vectors encoding corresponding genes. The transfected cells were cultured in shaker incubator at 120 rpm, 37°C, 9% CO2 for 4 to ~5 days. Culture supernatants were harvested and filtered, and proteins were purified through a Hispur Ni-NTA resin (Thermo Scientific, #88221) and following a Hiload 16/600 Superdex 200 column (GE healthcare, #28989335) according to manufacturer’s instructions. The protein purity was confirmed by means of SDS–polyacrylamide gel electrophoresis (SDS-PAGE).

### Synthesis, cloning and expression of monoclonal antibodies

A19-46.1, A19-61.1, B1-182.1, and A23-58.1 were synthesized, cloned and expressed as an immunoglobulin G1 (IgG1) containing an HRV3C protease site as previously reported (*14*). For all other antibodies, variable lambda and kappa light chain sequences were human codon optimized, synthesized and cloned into CMV/R-based lambda or kappa chain expression vectors, as appropriate (Genscript). ADG2 was kindly provided by Dr. Laura M Walker (Adagio Therapeutics, Waltham, MA) (*28*) and LY-CoV1404 by Dr. Stefanie Žentelis, Dr Emilie Lameignere and Kathryn Westendorf, MSc (AbCellera, Canada) (*29*). Previously published antibody vectors for LY-COV555 were used (*31*). For antibodies where vectors were unavailable (e.g., S309, CB6, REGN10933, REGN10987, COV2-2196, COV2-2130, CT-P59, C144, C135, S2E12) (*12*, *13*, *26*, *27*, *30*, *32*–*36*), published amino acids sequences were used for synthesis and cloning into corresponding pVRC8400 vectors (Genscript) (*56*, *57*). For antibody expression, equal amounts of heavy and light chain plasmid DNA were transfected into using Expi293 cells (Gibco, #A14527 by using Expi293 transfection reagent (Gibco, #A14525). The transfected cells were cultured in shaker incubator at 120 rpm, 37°C, 9% CO_2_ for 4 to ~5 days. Culture supernatants were harvested and filtered, mAbs were purified over Protein A (Cytiva, #GE17-1279-03) columns. Each antibody was eluted with IgG elution buffer (Pierce, #21009) and immediately neutralized with one tenth volume of 1M Tris-HCL pH 8.0. The antibodies were then buffer exchanged as least twice in PBS by dialysis.

### Full-length S constructs

Codon optimized cDNAs encoding full-length S from SARS CoV-2 (GenBank ID: QHD43416.1) were synthesized, cloned into the mammalian expression vector VRC8400 (*56*, *57*) and confirmed by sequencing. S containing D614G amino acid change was generated using the wt S sequence. Other variants containing single or multiple aa changes in the S gene from the S wt or D614G were made by mutagenesis using QuickChange lightning Multi Site-Directed Mutagenesis Kit (Agilent, #210515,) or via synthesis and cloning (Genscript). The S variants tested are B.1.351 (L18F, D80A, D215G, (L242-244)del, R246I, K417N, E484K, N501Y, A701V), P.1 (L18F, T20N, P26S, D138Y, R190S, K417T, E484K, N501Y, D614G, H655Y, T1027I, V1176F), B.1.1.7 (H69del, V70del, Y144del, N501Y, A570D, D614G, P681H, T716I, S982A, D1118H), B.1.617.2 (T19R, G142D, E156del, F157del, R158G, L452R, T478K, D614G, P681R, D950N), B.1.1.529 (A67V, H69del, V70del, T95I, G142D, V143del, Y144del, Y145del, N211del, L212I, ins214EPE, G339D, S371L, S373P, S375F, K417N, N440K, G446S, S477N, T478K, E484A, Q493R, G496S, Q498R, N501Y, Y505H, T547K, D614G, H655Y, N679K, P681H, N764K, D796Y, N856K, Q954H, N969K, L981F). The S genes containing single RBD amino acid changes from the B.1.1.529 variant were generated based on D614G construct by mutagenesis. These full-length S plasmids were used for pseudovirus production and for cell surface binding assays.

### Generation of 293 Flpin-TMPRSS2-ACE2 cell line

293 Flpin-TMPRSS2-ACE2 isogenic cell line was prepared by co-transfecting pCDNA5/FRT plasmid encoding TMPRSS2-T2A-ACE2 and pOG44 plasmid encoding Flp recombinase in 293 Flpin parental cell line (Thermo Fisher, #R75007). Cells expressing TMPRSS2-ACE2 were selected using Hygromycin (Thermo Fisher, #10687010) at 100 micrograms/ml. TMPRSS2 and ACE2 expression profiles in 293 Flpin-TMPSS2-ACE2 were characterized by flow cytometry using a mouse monoclonal antibody against TMPRSS2 (MillliporeSigma, #MABF2158-100UG) followed by an anti-mouse IgG1 APC conjugate (Jackson Laboratories, #115135164) and a molecular probe containing the SARS-CoV-2 receptor binding domain tagged with biotin (Sino Biological, $40592-V08B-B) followed by staining with a BV421 conjugated streptavidin probe (BD Biosciences, #405225).

### Pseudovirus neutralization assay

S-containing lentiviral pseudovirions were produced by co-transfection of packaging plasmid pCMVdR8.2, transducing plasmid pHR’ CMV-Luc, a TMPRSS2 plasmid and S plasmids from SARS CoV-2 variants into 293T cells using Lipofectamine 3000 transfection reagent (ThermoFisher Scientific, # L3000-001) (*58*, *59*). 293T-ACE2 cells (provided by Dr. Michael Farzan) or 293 flpin-TMPRSS2-ACE2 cells were plated into 96-well white/black Isoplates (PerkinElmer, #6005068) at 75,00 cells per well the day before infection of SARS CoV-2 pseudovirus. Serial dilutions of mAbs were mixed with titrated pseudovirus, incubated for 45 minutes at 37°C and added to cells in triplicate. 293 flpin-TMPRSS2-ACE2 cells were used for some of Class III antibodies like S309 and COV2-2130 while 293T-ACE2 cells were used for the rest of antibodies. Following 2 hours of incubation, wells were replenished with 150 ml of fresh media. Cells were lysed 72 hours later, and luciferase activity was measured with Microbeta (Perking Elmer, #2450-0120). Percent neutralization and neutralization IC_50_ and IC_80_ were calculated using GraphPad Prism 8.0.2.

### Cell surface binding

HEK293T cells were transiently transfected with plasmids encoding full length SARS CoV-2 spike variants using lipofectamine 3000 (ThermoFisher, # L3000-001) following manufacturer’s protocol. After 40 hours, the cells were harvested and incubated with monoclonal antibodies (0.5 μg/ml) or biotinylated-human ACE2 (Acro Biosystems, AC2-H82F9) for 30 minutes. After incubation with the antibodies or ACE2, the cells were washed and incubated with an allophycocyanin conjugated anti-human IgG (Jackson Immunoresearch Laboratories, #709-136-149) or BV421 conjugated streptavidin conjugate for another 30 minutes. The cells were then washed and fixed with 1% paraformaldehyde (Electron Microscopy Sciences, #15712-S). The samples were then acquired in a BD LSRFortessa X-50 flow cytometer (BD biosciences) and analyzed using Flowjo (BD biosciences). The concentration of ACE2, 10 μg/ml, was determined empirically by titration on WA-1 spike expressing cells. Spike expression level was determined using the SARS-CoV-2 S2 antibody, WS6, that binds to a conserved epitope in the stem-helix in each of the variants (*60*). A variant spike protein expression adjustment factor (A_variant_) was calculated by dividing the mean fluorescent intensity (MFI) for WS6 antibody binding of a variant S by the MFI of WS6 binding to D614G S. Relative binding for antibodies or ACE2 was calculated with the following formula

$$\text{Ligand relative binding = }\frac{A_{\text{variant}}\;\times \;\left(\text{MFI ligand to variant}\right)}{A_{\text{D614G}}\;\times \;\left(\text{MFI ligand to variant}\right)}\times \text{ 100\%}$$

where ligand is an antibody or ACE2 and variant is D614G, B.1.1.7, B.1.351, P.1, B.1.617.2 or B.1.1.529. The adjustment factor for the sham transfected cells was set to 1.

### Production of Fab fragments from monoclonal antibodies

To generate mAb-Fab, IgG was incubated with HRV3C protease (EMD Millipore, #71493) at a ratio of 100 units per 10 mg IgG with HRV 3C Protease Cleavage Buffer (150 mM NaCl, 50 mM Tris-HCl, pH 7.5) at 4°C overnight. Fab was purified by collecting flowthrough from Protein A column (GE Health Science), and Fab purity was confirmed by SDS-PAGE.

### Determination of binding kinetics of ACE2

Binding kinetics and affinities of ACE2 to SARS-CoV-2 S2P variants were assessed by surface plasma resonance on a Biacore S-200 (GE Healthcare) at 25^o^C in the HBS-EP+ buffer (10 mM HEPES, pH 7.4, 150 mM NaCl, 3 mM EDTA, and 0.05% surfactant P20). Fc-reactive anti-human IgG antibody (Cytiva, #BR100839) was coupled to a CM5 chip to approximately 10,000 RU, and dimeric, Fc-tagged ACE2 (ACRO Biosystems, AC2-H82F9) at 35 μg/ml was captured for 60 seconds at 10 μl/min to a response of approximately 200 RU. Serially diluted SARS-CoV-2 S2P variants starting at 100 nM were flowed through the sample and reference channels for 180 seconds at 30 μl/min, followed by a 300 second dissociation phase at 30 uL/min. The chip was regenerated using 3 M MgCl_2_ for 30 seconds at 50 μl/min. Blank sensorgrams were obtained with HBS-EP+ buffer. Blank-corrected sensorgrams of the S2P concentration series were fitted globally with Biacore S200 evaluation software using a 1:1 model of binding. Plots were generated using GraphPad Prism.

### Cryo-EM specimen preparation and data collection

Cryo-EM grids for the B.1.1.529 spike stabilized with the “2P” mutations were prepared at 0.5 mg/ml in a buffer containing 10 mM HEPES, pH 7.5 and 150 mM NaCl. For the spike-Fab complexes, the stabilized SARS-CoV-2 spikes of B.1.1.529 or WA-1were1. were mixed with Fab or Fab combinations at a molar ratio of 1.2 Fab per protomer in PBS with final spike protein concentration at 0.5 mg/ml. n-Dodecyl β-D-maltoside (DDM) detergent was added to the protein complex mixtures shortly before vitrification to a concentration of 0.005%. Quantifoil R 2/2 gold grids were subjected to glow discharging in a PELCO easiGlow device (air pressure: 0.39 mBar, current: 20 mA, duration: 30 s) immediately before specimen preparation. Cryo-EM grids were prepared using an FEI Vitrobot Mark IV plunger with the following settings: chamber temperature of 4°C, chamber humidity of 95%, blotting force of –5, blotting time of 2 to 3.5 s, and drop volume of 2.7 µl. Datasets were collected at the National CryoEM Facility (NCEF), National Cancer Institute, on a Thermo Scientific Titan Krios G3 electron microscope equipped with a Gatan Quantum GIF energy filter (slit width: 20 eV) and a Gatan K3 direct electron detector (table S2). Four movies per hole were recorded in the counting mode using Latitude software. The dose rate was 14.65 e-/s/pixel.

### Cryo-EM data processing and model fitting

Data process workflow, including motion correction, CTF estimation, particle picking and extraction, 2D classification, ab initio reconstruction, homogeneous refinement, heterogeneous refinement, non-uniform refinement, local refinement and local resolution estimation, were carried out with C1 symmetry in cryoSPARC 3.3 (*61*). The overall resolution was 3.29 Å for the map of B.1.1.529 spike alone structure, 3.85 Å for the map of B.1.1.529 spike in complex with A19-46.1, 2.83 Å for the map of WA-1 spike in complex with A19-61.1 and B1-182.1, and 3.86Å for the map of B.1.1.529 spike in complex with A19-46.1 and B1-182.1. The coordinates of the SARS-CoV-2 spike and Fab B1-182.1 in PDB ID: 7MM0 were used as initial models for fitting the cryo-EM maps. Outputs from AlphaFold 2.0 modelling were used as initial models for Fab A19-46.1 and Fab A19-61.1. To resolve the RBD-antibody interface, local refinements were performed, a mask for the entire spike-antibody complex without the RBD-antibody region was used to extract the particles and a mask encompassing the RBD-antibody region was used for refinement. Local refinements of the Fab A19-46.1 and B.1.1.529 RBD interface and the Fab A19-46.1, Fab B1-182.1 and B.1.1.529 RBD interface resulted 4.68 Å and 4.83 Å maps, respectively, which enabled the definition of the backbone. However, the side chains were not fully resolved. Iterative manual model building and real-space refinement were carried out in Coot (*48*) and in Phenix (*62*), respectively. Molprobity (*63*) was used to validate geometry and check structure quality at each iteration step. UCSF Chimera and ChimeraX were used for map fitting and manipulation (*64*).

### Differential scanning calorimetry (DSC)

DSC measurements were performed using a VP-ITC (Microcal) instrument. Spike samples were diluted to 0.125mg/ml in PBS and scanned from 20 to 95°C at a rate of 1°C per minute. Thermal denaturation (*T*_m_) temperature and total enthalpy of unfolding was calculated using the Microcal analysis system in Origin.

### Biolayer interferometry binding assay

The antibody binding panel was performed on a FortéBio Octet HTX instrument with black, tilted 384-well plates (Greiner Bio-One). All steps of pre-soaking, binding and dissociation were performed in PBS with 1% BSA at pH 7.4. IgGs and dACE2-Fc were loaded onto Anti-Human Fc Sensor Tips (FortéBio) at a concentration of 1-4μg/ml, resulting in a load response of 0.85-1.5 nm. The plates were agitated at 1,000 rpm and the experiment run at 30°C. Antibodies and ACE2 were loaded onto the tips for 2 minutes, bound to 100nM S2P protein for 5 minutes and dissociated in buffer for 5 minutes. Reference well subtraction was performed with the Data Analysis Software HT v12.0 (FortéBio). The graphs were generated in GraphPad Prism.

## Supplementary Material

20220701-1

## Supplementary Materials

### Other Supplementary Material for this manuscript includes the following:

MDAR Reproducibility Checklist
