# Supplementary material for: Structural basis for potent antibody neutralization of SARS-CoV-2 variants including B.1.1.529
Source: Science. 2022 Mar 24;376(6591):eabn8897. doi: 10.1126/science.abn8897 (PMC9580340; doi:10.1126/science.abn8897)
Supplement: Supplementary file 2 — Figs. S1 to S10 Tables S1 and S2 [file science.abn8897_sm.pdf]

Science AAAS

Supplementary Materials for

Structural basis for potent antibody neutralization of SARS-CoV-2 variants including B.1.1.529

Tongqing Zhou *et al*.

Corresponding authors: Tongqing Zhou, tzhou@nih.gov; John Misasi, john.misasi@nih.gov; Peter D. Kwong, pdkwong@nih.gov

Science **376**, eabn8897 (2022)
DOI: 10.1126/science.abn8897

The PDF file includes:

Figs. S1 to S10
Tables S1 and S2

Other Supplementary Material for this manuscript includes the following:

MDAR Reproducibility Checklist

Table S1. Cryo-EM Data Collection, Refinement and Validation Statistics for SARS COV-2 Spike and Antibody Complexes.

	SARS-CoV-2 Omicron spike (EMD-25792) (PDB 7TB4)	SARS-CoV-2 Omicron spike in complex with A19-46.1 (EMD-25807) (PDB 7TCA)	SARS-CoV-2 Omicron spike in complex with A19-46.1 after local refinement (EMD-25806) (PDB 7TC9)	SARS-CoV-2 Omicron spike in complex with A19-46.1 and B1-182.1 (EMD-25808) (PDB 7TCC)	SARS-CoV-2 Omicron spike in complex with B1-182.1 and A19-46.1 after local refinement (EMD-26256) (PDB 7U0D)	SARS-CoV-2 spike in complex with B1-182.1 and A19-61.1 (EMD-25794) (PDB 7TB8)	B1-182.1:A19-61.1:RBD complex after local refinement (EMD-25797) (PDB 7TBF)
**Data collection and processing**							
Magnification	105,000	105,000		105,000		105,000	
Voltage (kV)	300	300		300		300	
Electron exposure ($e^-/Å^2$)	40.0	40.0		40.0		40.0	
Defocus range (μm)	-1.0 to -2.5 μm	-1.0 to -2.5 μm		-1.0 to -2.5 μm		-1.0 to -2.5 μm	
Pixel size (Å)	0.855	0.855		0.855		0.873	
Symmetry imposed	C1	C1		C1		C1	
Final particle images (no.)	266434	79077		46244		358526	
Map resolution (Å)	3.29	3.85	4.68	3.86	4.83	2.83	3.10
FSC threshold	0.5	0.5		0.5		0.5	0.5
**Refinement**							
Initial model used (PDB code)	7MM0	7TB4	7TB4	7TB4	7MM0	7MM0	7TB8
Model resolution (Å)	3.15	3.29	3.29	3.29	3.15	3.15	2.83
FSC threshold	0.5	0.5	0.5	0.5	0.5	0.5	0.5
Map sharpening *B* factor ($Å^2$)	-130.7	-94.1	-282	-70.5	-171.4	-61.8	-46.5
Model composition							
Non-hydrogen atoms	26899	33706	4298	37713	8188	33230	6617
Protein residues	3329	4244	642	4749	1071	4189	857
Ligands	59	56	1	57	1	43	2
B factors ($Å^2$)(mean)							
Protein	97.2	214.4	178.0	74.3	190.4	76.0	51.1
Ligand	97.4	151.5	154.5	170.39	96.4	60.0	52.0
R.m.s. deviations							
Bond lengths (Å)	0.005	0.002	0.006	0.006	0.019	0.003	0.007
Bond angles (°)	0.655	0.587	0.828	0.742	0.857	0.620	0.966
Validation							
MolProbity score	1.95	1.97	2.39	2.35	2.34	1.86	2.11
Clash score	7.9	9.7	21.1	18.8	20.0	7.3	8.3
Poor rotamers (%)	0.1	0.03	0	0.02	0	0	0
Ramachandran plot							
Favored (%)	91.2	92.6	89.3	88.9	90.1	92.8	85.0
Allowed	8.5	7.1	10.1	10.8	9.7	6.9	13.9
Disallowed	0.3	0.3	0.6	0.3	0.2	0.3	1.2

Table S2. B.1.1.529 mutation introduced hydrogen bonds and salt bridges.

Hydrogen bonds			
**##**	**Protomer 1**	**Dist. [Å]**	**Protomer 2**
1	C:SER 982[OG	2.51	**B:LYS 547[NZ]**
2	**C:LYS 856**[NZ]	2.60	B:THR 572[OG1]
3	**B:LYS 764**[NZ]	2.78	A:GLN 314[OE1]
4	**B:LYS 856**[NZ]	2.99	A:THR 572[OG1]
6	**C:LYS 547[NZ]**	3.89	A:ASN 978[OD1]
**Salt bridges**			
**##**	**Protomer 1**	**Dist. [Å]**	**Protomer 2**
1	**B:LYS 856[NZ]**	3.94	A:ASP 568[OD2]
2	C:ASP 571[OD2]	3.42	**A:LYS 856[NZ]**

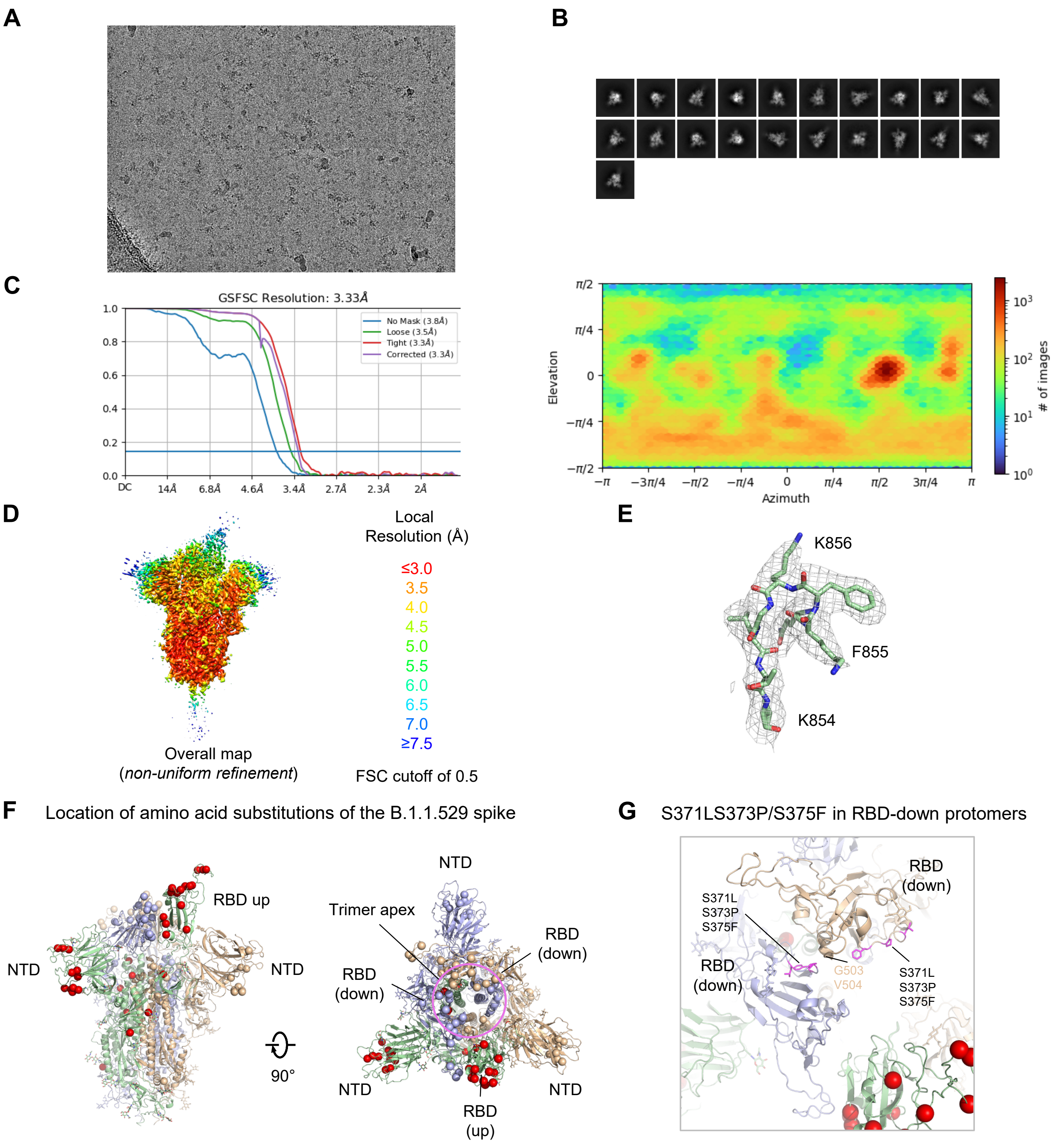

Figure S1. Cryo-EM details of the SARS-CoV-2 B.1.1.529 spike.

A. Representative micrograph.

B. Representative 2D class averages are shown.

C. The gold-standard Fourier shell correlation resulted in a resolution of 3.33 Å for the overall map using non-uniform refinement with C1 symmetry (left panel); the orientations of all particles used in the final refinement are shown as a heatmap (right panel).

D. The local resolution of the final overall map is shown contoured at 0.373 (5.7σ). Resolution estimation was generated through cryoSPARC using an FSC cutoff of 0.5.

E. Representative density is shown for the Lys856 region where B.1.1.529 mutated. The contour level is 5σ.

F. Location of the B.1.1.529 amino acid substitutions. The residues are shown as spheres of their respective Cα-atoms.

G. Positions of the S371LS373P/S375F substitutions in the two RBDs in down conformation shown in the same top-view orientation as in panel F.

A

Domains and amino acid substitutions of the B.1.1.529 spike

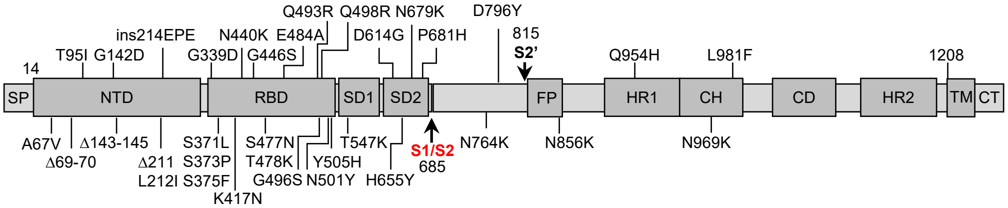

B

Differential scanning calorimetry of SARS-CoV-2 spike

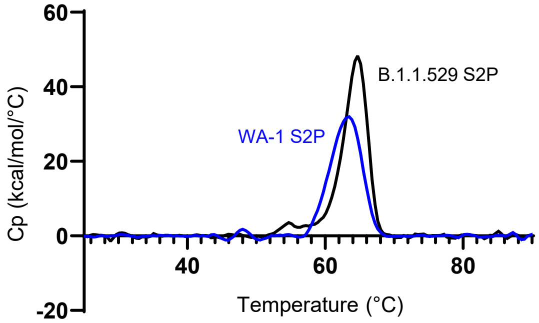

Sample	DH (kcal/mol)	High Temp Tm (°C)
WA-1 S2P	179	63.80
B.1.1.529 S2P	222	64.72

Figure S2. Domain arrangement and amino acid substitutions of the B.1.1.529 spike and differential scanning calorimetry of SARS-CoV-2 spikes (S2P) for WA-1 and B.1.1.529 variants.

A. Linear mapping of the domains and amino acid substitutions of the B1.1.529 spike. The cleavage location between the S1 and S2 subunits is shown in red. SP: signal peptide, NTD: N-terminal domain, RBD: receptor binding domain. SD: subdomain, FP: fusion peptide, HR1: heptad repeat 1, CH: central helix, CD: connector domain, HR2: heptad repeat 2, TM: transmembrane domain, CT: cytoplasmic tail. Vertical lines indicated the relative locations of amino acid substitutions in B.1.1.529 spike.

B. Differential scanning calorimetry of SARS-CoV-2 spikes.

A

ACE2 cell surface binding to variant spike proteins

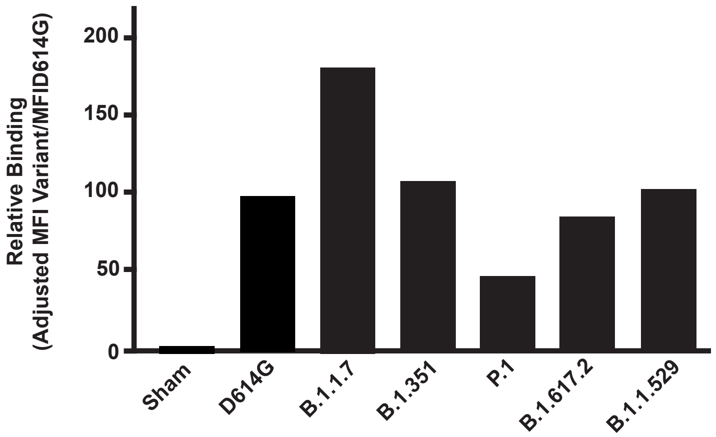

B Raw and fitted sensorgrams of surface plasmon resonance for dimeric ACE2 binding to SARS-CoV2 spike proteins

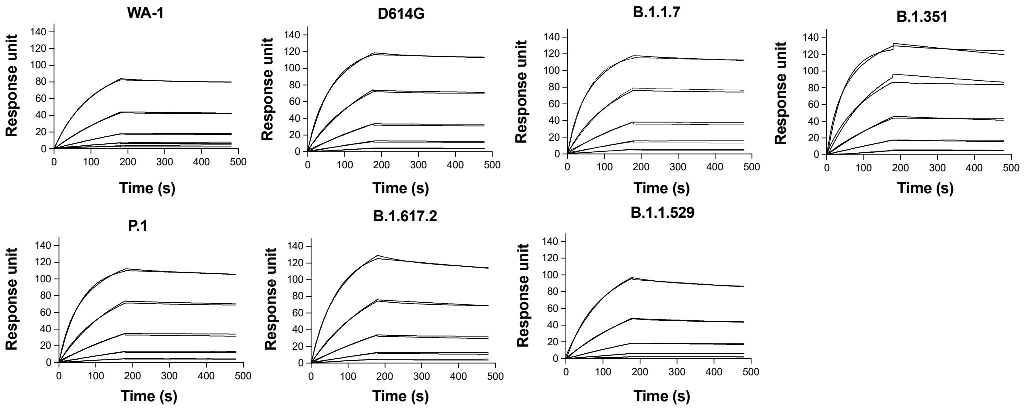

C Kinetics of ACE2 binding to SARS-CoV2 spike proteins measured by surface plasmon resonance

Variant	ka (1/Ms)	kd (1/s)	Kapp (M)	Rmax (RU)	Chi^2 (RU^2)
WA-1	9.78E+04	1.12E-04	1.14E-09	100.2	1.01
D614G	1.48E+05	1.08E-04	7.27E-10	125.9	1.18
B.1.1.7	1.78E+05	1.05E-04	5.87E-10	121.0	2.81
B.1.351	2.09E+05	3.44E-04	1.70E-09	138.7	115
P.1	1.68E+05	1.43E-04	8.50E-10	116.6	1.55
B.1.617.2	1.36E+05	3.23E-04	2.37E-09	139.8	1.45
B.1.1.529	8.47E+04	3.22E-04	3.80E-09	123.4	0.24

Figure S3. Monomeric ACE2 binding to SARS-CoV-2 B.1.1.529 cell surface expressed spike and S-2P trimer.

A. Full length spike proteins from the indicated SARS-CoV-2 variants were expressed on the surface of transiently transfected 293T cells and binding to dimeric human ACE2 was assessed by flow cytometry. ACE2 MFI binding signal was adjusted based upon spike protein expression level (See Figure S4). Shown is the ratio of the adjusted mean fluorescence intensity (MFI) of ACE2 to the indicated spike expressing cells to the adjusted MFI of ACE2 bound to D614G spike expressing cells. The data is expressed as a percentage and shown as the average of two independent experiments.
B. Raw and fitted binding curves for dimeric human ACE2 protein to 2-proline stabilized spike proteins for each of the indicated variants.
C. Kinetic parameters with affinity of trimeric spike binding to dimeric ACE2 expressed as apparent affinity. Rmax and Chi^2 values were obtained using the curves in panel B.

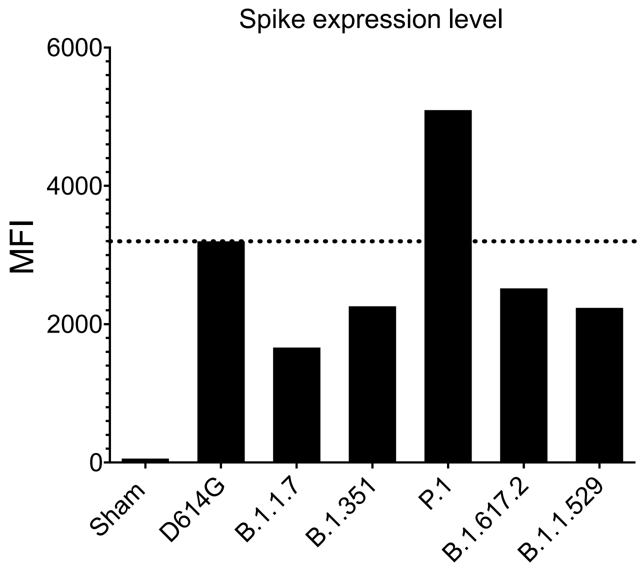

Figure S4. Evaluation of SARS-CoV-2 spike expression level by flow cytometry.
Full length spike proteins from the indicated SARS-CoV-2 variants were expressed on the surface of transiently transfected 293T cells and spike protein expression level was measured using the SARS-CoV-2 antibody WS6, which targets a conserved portion of the S2 stem-helix. Shown is a representative experiment (n=2).

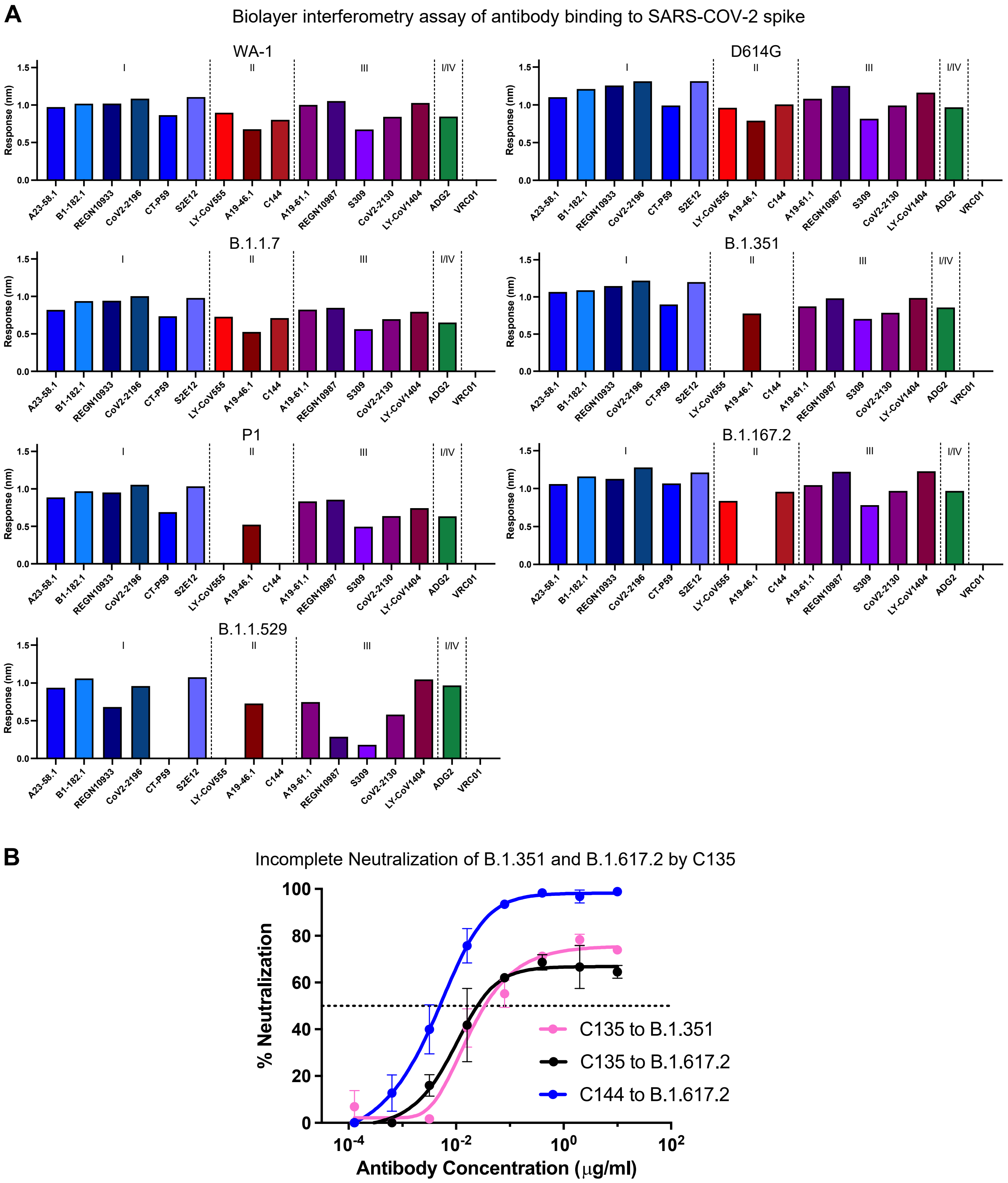

Figure S5. Antibody binding to S2P spike proteins and Neutralization of B.1.351 and B.1.617.2 by C135.

A. Biolayer interferometry assay of antibody binding to purified spike proteins of SARS-CoV-2 variants. Antibodies are. Grouped by the Barnes Class I-IV.

B. Lentiviruses pseudotyped with SARS-CoV-2 spike proteins from B.1.351 or B.1.617.2 were incubated with serial dilutions of the indicated concentrations of either C135 or C144 and %neutralization measured.

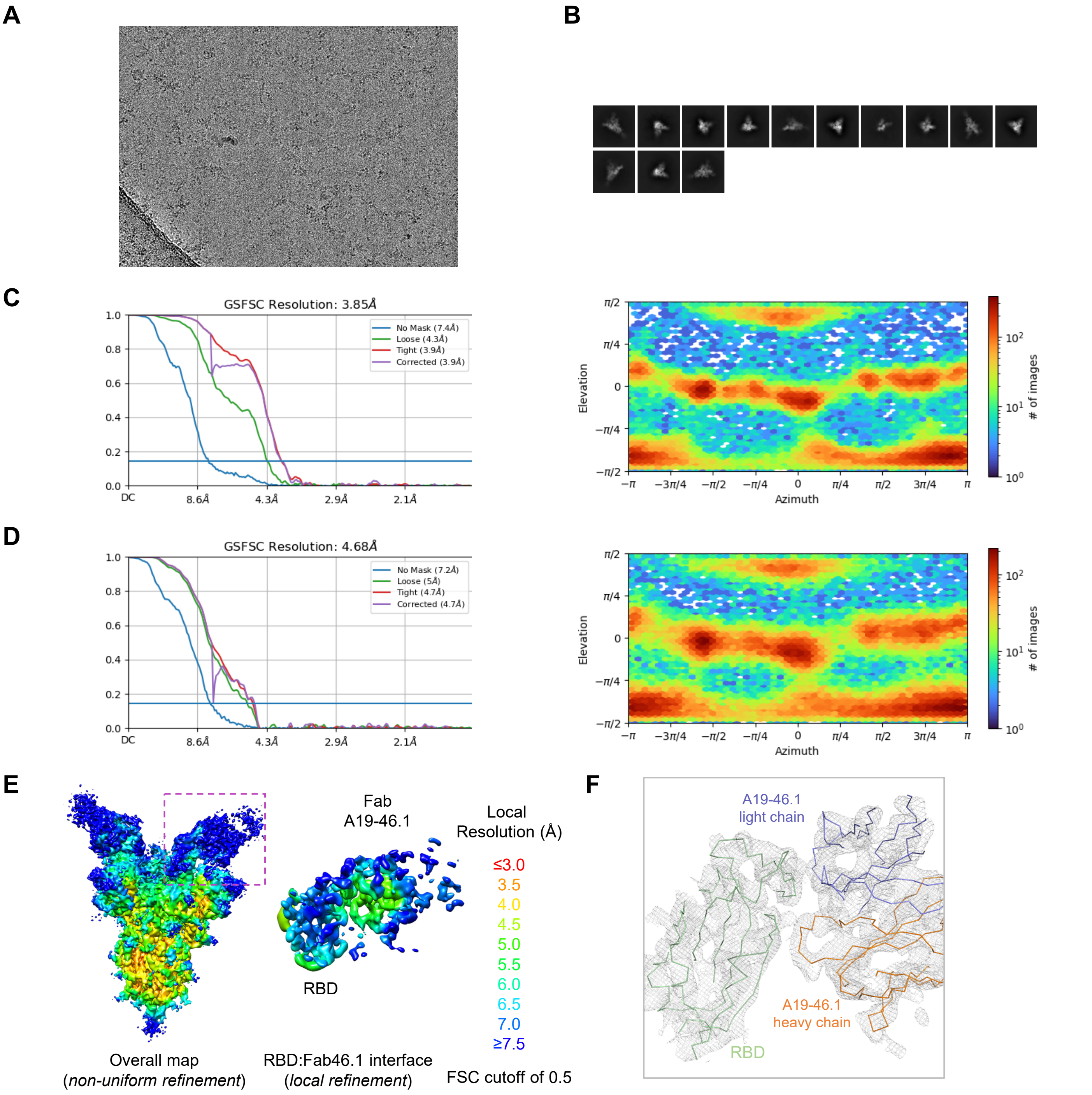

Figure S6. Cryo-EM details of A19-46.1 Fab in complex with SARS-CoV-2 B.1.1.529 spike.

A. Representative micrograph.

B. Representative 2D class averages.

C. The gold-standard Fourier shell correlation resulted in a resolution of 3.85 Å for the overall map using non-uniform refinement with C1 symmetry (left panel); the orientations of all particles used in the final refinement are shown as a heatmap (right panel).

D. The gold-standard Fourier shell correlation resulted in a resolution of 4.68 Å for the masked local refinement of the RBD:A19-46.1 interface (left panel) obtained using particle subtraction followed by local refinement; the orientations of all particles used in the local refinement are shown as a heatmap (right panel).

E. The local resolution of the final overall map and locally refined map is shown contoured at 0.068 (4.5σ) and 0.417 (21.5σ), respectively. Resolution estimation was generated through cryoSPARC using an FSC cutoff of 0.5.

F. Local density is shown for the antibody-RBD interface. The contour level is 1.5σ.

A S309 neutralization of SARS-CoV-2 with single B.1.1.529 RBD amino acid substitutions

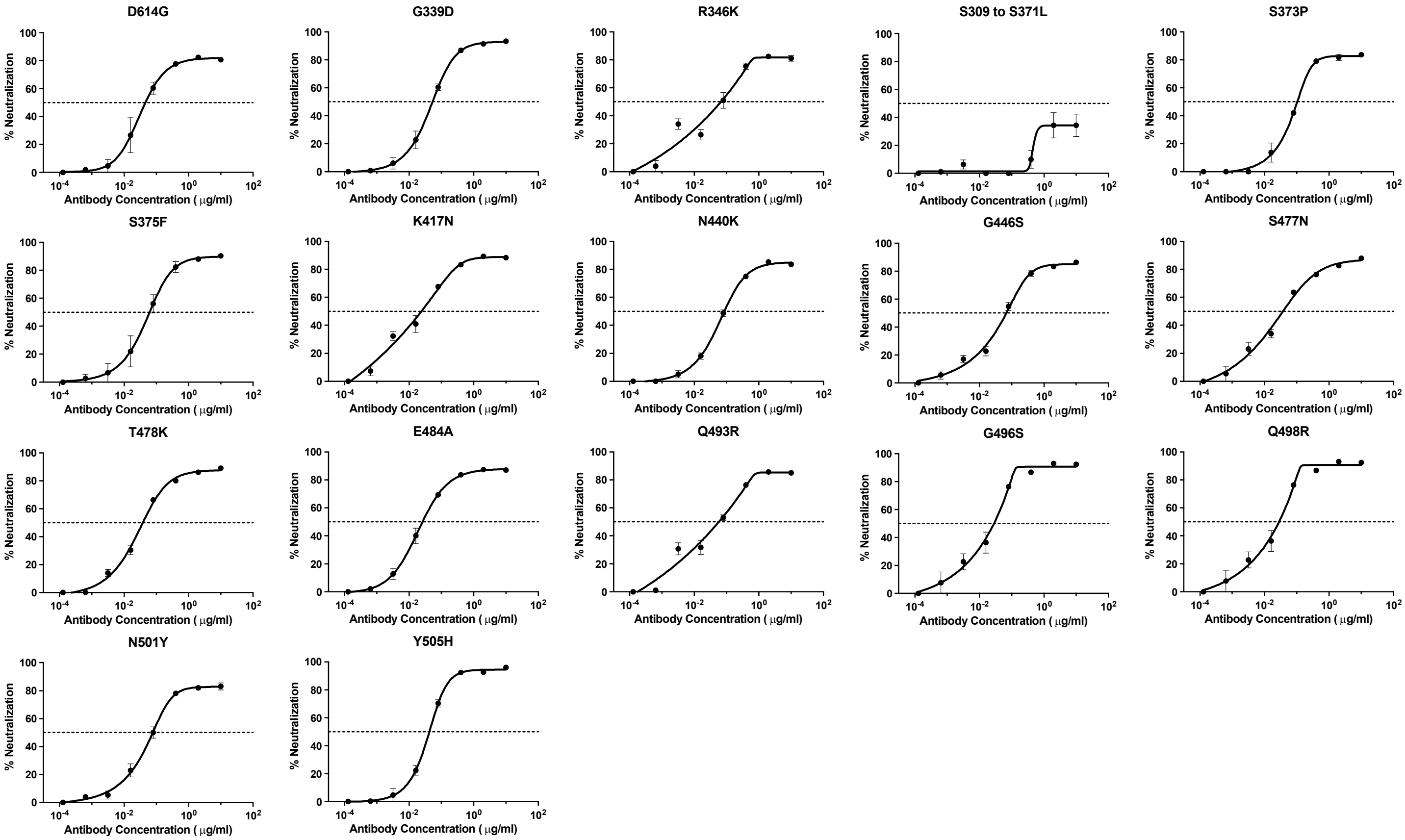

B S371L neutralization by select Class I, II and III antibodies

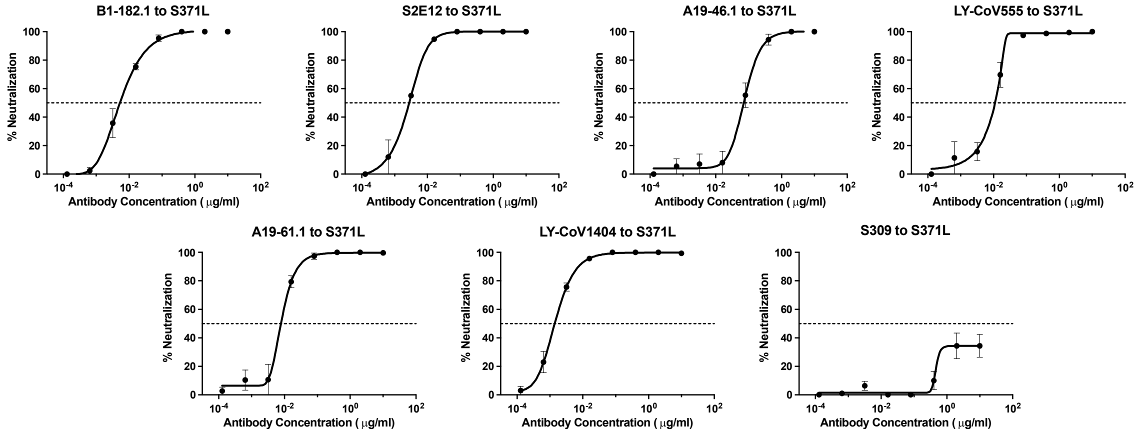

Figure S7. SARS-CoV-2 neutralization curves.

A. Lentiviruses pseudotyped with SARS-CoV-2 spike proteins containing either D614G or D614G plus single amino acid changes found in the RBD of B.1.1.529 were incubated with serial dilutions of S309 using 293 flpin-TMPRSS2-ACE2 cells. Experiments is representative of two independent experiments with 3 replicates per assay.

B. Lentiviruses pseudotyped with SARS-CoV-2 spike proteins containing D614G plus S371L were incubated with serial dilutions of the indicated antibodies. S309 was tested on 293 flpin-TMPRSS2-ACE2 cells while all the other antibodies were tested on 293T-ACE2 cells. Experiments is representative of two independent experiments with 3 replicates per assay.

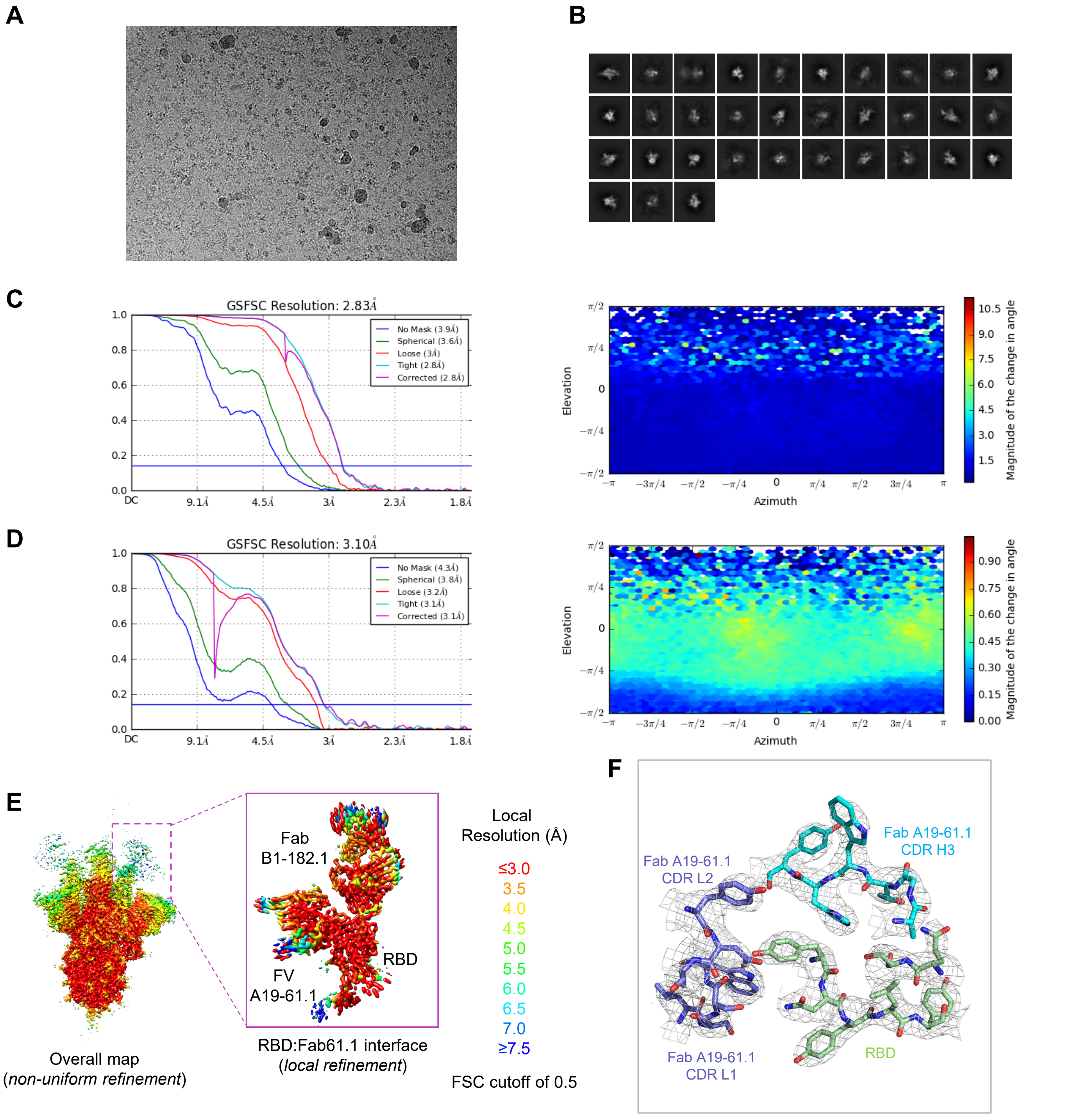

Figure S8. Cryo-EM details of A19-61.1 Fab and Fab B1-182.1 in complex with SARS-CoV-2 WA-1 spike.

A. Representative micrograph.

B. Representative 2D class averages are shown.

C. The gold-standard Fourier shell correlation resulted in a resolution of 2.83 Å for the overall map using non-uniform refinement with C1 symmetry (left panel); the orientations of all particles used in the final refinement are shown as a heatmap (right panel).

D. The gold-standard Fourier shell correlation resulted in a resolution of 3.1 Å for the masked local refinement of the RBD:A19-61.1-B1-182.1 ternary interface (left panel) obtained using particle subtraction followed by local refinement; the orientations of all particles used in the local refinement are shown as a heatmap (right panel).

E. The local resolution of the final overall map and locally refined map is shown contoured at 0.154 (5.7σ) and 0.246 (22.2σ), respectively. Resolution estimation was generated through cryoSPARC using an FSC cutoff of 0.5.

F. Representative density is shown for the RBD:A19-61.1 interface after local refinement indicating well-defined side chains. The contour level is 10σ.

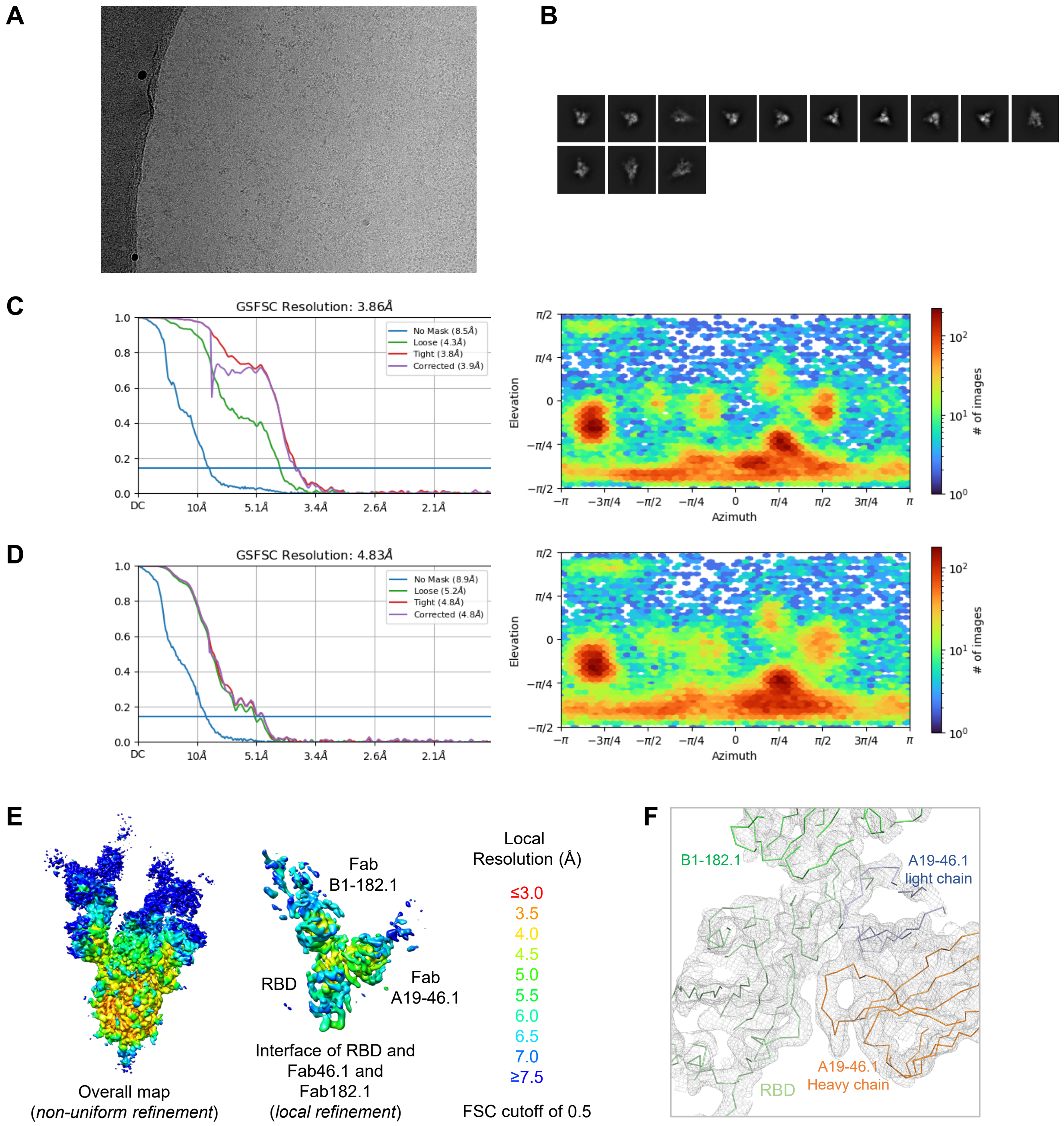

Figure S9. Cryo-EM details of A19-46.1 Fab and Fab B1-182.1 in complex with SARS-CoV-2 B.1.1.529 spike.

A. Representative micrograph.

B. Representative 2D class averages are shown.

C. The gold-standard Fourier shell correlation resulted in a resolution of 3.85 Å for the overall map using non-uniform refinement with C1 symmetry (left panel); the orientations of all particles used in the final refinement are shown as a heatmap (right panel).

D. The gold-standard Fourier shell correlation resulted in a resolution of 4.83 Å for the RBD and antibody interface; the orientations of all particles used in the final refinement are shown as a heatmap (right panel).

E. The local resolution of the final overall map and the interface between Fab B1-182.1, Fab A19-46.1 and RBD are shown contoured at 0.25 (8.5σ) and 0.406 (23.2σ), respectively. Resolution estimation was generated through cryoSPARC using an FSC cutoff of 0.5.

F. Overall density is shown for the B.1.1.529 RBD and Fab 19-46.1 interface with mainchain traces. The contour level is 1.5σ.

A

Differences between B.1.1.529 (BA.1) and BA.2 spike protein

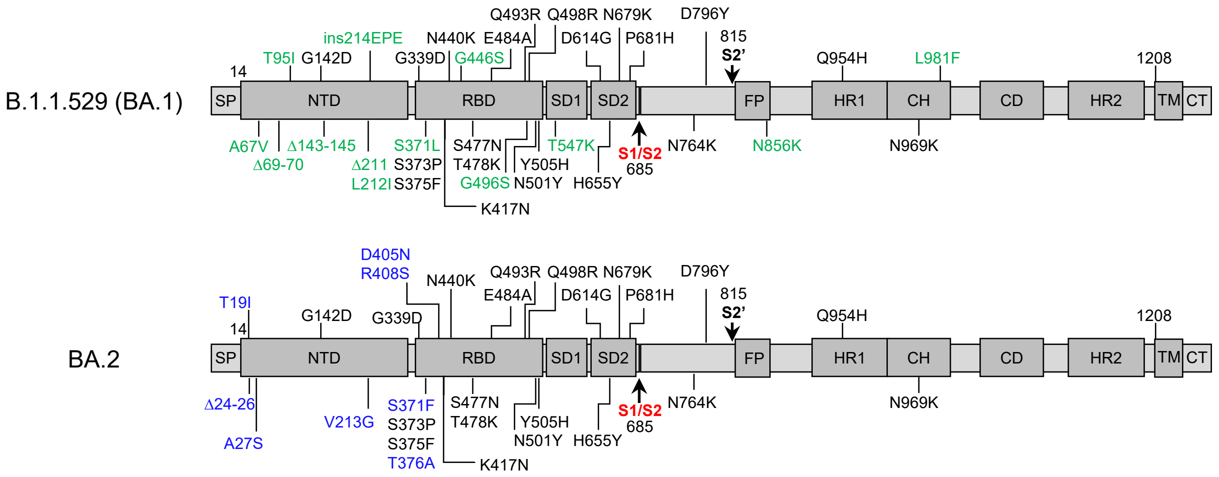

B

B1.1.529 (BA.1) and BA.2 variant neutralization of by monoclonal antibodies

Antibody	Generic name	Class	D614G IC50	D614G IC80	B.1.1.529 (BA.1) IC50	B.1.1.529 (BA.1) IC80	BA.2 IC50	BA.2 IC80
A23-58.1	--	I	1.3	4.5	231	1132	264	896
B1-182.1	--	I	0.9	2.4	281	1301	244	1037
COV2-2196	tixagevimab *	I	2.0	3.2	269	900	571	3769
S2E12	--	I	1.4	2.9	38.1	112	9.7	43.7
CB6	etesevimab	I	50.5	108.9	> 10,000	> 10,000	> 10,000	> 10,000
REGN10933	casirivimab	I	6.1	16.0	> 10,000	> 10,000	> 10,000	> 10,000
CT-P59	regdanvimab	I	1.5	4.6	> 10,000	> 10,000	> 10,000	> 10,000
ADG2	--	I-IV	5.1	14.7	2037	8113	> 10,000	> 10,000
A19-46.1	--	II	19.4	40.6	223	376	103	320
LY-COV555	Bamlanivimab	II	3.6	10.4	> 10,000	> 10,000	> 10,000	> 10,000
C144	--	II	5.1	9.8	> 10,000	> 10,000	> 10,000	> 10,000
A19-61.1	--	III	7.7	20.1	> 10,000	> 10,000	8.1	24.0
REGN10987	imdevimab	III	20.0	412	> 10,000	> 10,000	> 10,000	> 10,000
COV2-2130	cilgavimab *	III	3.7	10.9	5850	> 10,000	5.1	15.9
C135	--	III	10.8	64.3	> 10,000	> 10,000	> 10,000	> 10,000
S309	Sotrovimab *	III	36.1	163	281	1336	1374	> 10,000
LY-CoV1404	Bebtelovimab	III	3.0	5.8	5.1	14.4	0.6	2.5

C

B1.1.529 (BA.1) and BA.2 variant neutralization of by antibody combinations

Antibody	Class	D614G IC50	D614G IC80	B.1.1.529 (BA.1) IC50	B.1.1.529 (BA.1) IC80	BA.2 IC50	BA.2 IC80
CB6/LY-CoV555	I/II	5.6	14.1	> 10,000	> 10,000	> 10,000	> 10,000
REGN10933/REGN10987	I/III	3.4	6.3	> 10,000	> 10,000	> 10,000	> 10,000
COV2-2196/COV2-2130	I/III	2.2	4.1	50.8	131	3.6	11.5
B1-182.1/A19-46.1	I/II	1.6	2.7	28.3	65.7	24.1	77.0
B1-182.1/A19-61.1	I/III	1.8	3.9	311	426	7.4	23.6
B1-182.1/S309	I/III	0.3	2.5	58.1	194	148	390

Figure S10. SARS-CoV-2 variant BA.2 spike protein substitutions and antibody neutralization.

A. Linear mapping of the domains and amino acid substitutions of the B1.1.529 (BA.1) and BA.2 spike. The cleavage location between the S1 and S2 subunits is shown in red. SP: signal peptide, NTD: N-terminal domain, RBD: receptor binding domain. SD: subdomain, FP: fusion peptide, HR1: heptad repeat 1, CH: central helix, CD: connector domain, HR2: heptad repeat 2, TM: transmembrane domain, CT: cytoplasmic tail. Vertical lines indicated the relative locations of amino acid substitutions in the spike protein, black substitutions are common, green are only in B.1.1.529 and blue are only in BA.2.

B-C. Lentiviruses pseudotyped with SARS-CoV-2 spike from B.1.1.529 (BA.1) or BA.2 were incubated with serial dilutions of the indicated antibodies or antibody combinations, and IC_{50} and IC_{80} values determined. S309 was tested on 293 flpin-TMPRSS2-ACE2 cells while all the other antibodies were tested on 293T-ACE2 cells. Ranges are indicated with white (>10,000 ng/ml), light blue (>1000 to ≤10,000 ng/ml), yellow (>100 to ≤1000 ng/ml), orange (>50 to ≤100 ng/ml), red (>10 to ≤50 ng/ml), maroon (>1 to ≤10 ng/ml), and purple (≤1 ng/ml). When available generic names of antibodies under therapeutic investigation are shown. Grey shading indicates antibodies that previously or currently have received Emergency Use Authorization from the United States Food and Drug Administration. Generic names with an * indicates that the therapeutic antibody products with the same binding regions as the antibodies being tested but contain amino acid changes in their Fc domains which extend their half-life.
